# Intracellular Dynamics in Cuneate Nucleus Neurons Support Self-Stabilizing Learning of Generalizable Tactile Representations

**DOI:** 10.3389/fncel.2018.00210

**Published:** 2018-07-31

**Authors:** Udaya B. Rongala, Anton Spanne, Alberto Mazzoni, Fredrik Bengtsson, Calogero M. Oddo, Henrik Jörntell

**Affiliations:** ^1^The BioRobotics Institute, Scuola Superiore Sant'Anna, Pisa, Italy; ^2^Section for Neurobiology, Department of Experimental Medical Sciences, Biomedical Center, Lund University, Lund, Sweden

**Keywords:** cuneate nucleus, neurophysiology, neuronal plasticity, Intrinsic dynamics, tactile, touch, synaptic integration

## Abstract

How the brain represents the external world is an unresolved issue for neuroscience, which could provide fundamental insights into brain circuitry operation and solutions for artificial intelligence and robotics. The neurons of the cuneate nucleus form the first interface for the sense of touch in the brain. They were previously shown to have a highly skewed synaptic weight distribution for tactile primary afferent inputs, suggesting that their connectivity is strongly shaped by learning. Here we first characterized the intracellular dynamics and inhibitory synaptic inputs of cuneate neurons *in vivo* and modeled their integration of tactile sensory inputs. We then replaced the tactile inputs with input from a sensorized bionic fingertip and modeled the learning-induced representations that emerged from varied sensory experiences. The model reproduced both the intrinsic membrane dynamics and the synaptic weight distributions observed in cuneate neurons *in vivo*. In terms of higher level model properties, individual cuneate neurons learnt to identify specific sets of correlated sensors, which at the population level resulted in a decomposition of the sensor space into its recurring high-dimensional components. Such vector components could be applied to identify both past and novel sensory experiences and likely correspond to the fundamental haptic input features these neurons encode *in vivo*. In addition, we show that the cuneate learning architecture is robust to a wide range of intrinsic parameter settings due to the neuronal intrinsic dynamics. Therefore, the architecture is a potentially generic solution for forming versatile representations of the external world in different sensor systems.

## Introduction

The problem of how to represent a complex external world to support non-trivial versatility of action has a deadening presence both in neuroscience (Loeb and Fishel, 2014; Spanne and Jorntell, 2015), robotics and artificial intelligence (AI) systems (Service, 2014). For neuroscience, the issue is closely associated with the understanding of the brain—without knowledge of how information of the world is represented in neuronal circuitry, it is difficult to decipher its functional mechanisms. An important related issue is how biological systems can generalize previously learnt sensory experiences to apply them to the interpretation of novel contexts—lack of generalization capability is a limitation in classical pattern recognition systems (Spanne and Jorntell, 2015), to which AI and DNN systems has an ancestry, and likely an important reason why such systems can be comparatively easily fooled (Nguyen et al., 2015). As versatility of interaction with the external world is a hallmark of brain function, an important question is how that versatility can be supported.

The skin is an interface that directly interacts with the physical world, using 10,000's of tactile sensors (Johansson and Flanagan, 2009). Current models of the organization of tactile neural systems to a large extent build on assumptions that the brain needs to identify edges, shapes or other physical parameters that human external observers may deem important to represent (Pei et al., 2011; Sathian, 2016). Using sparse-coding interpretations of neural coding combined with grandmother neuron-like theory (Barlow, 1972) such a system can be expected to work in the classification of a large range of tactile experiences. However, classifying systems based on pattern recognition can suffer from problems with generalization, i.e., where learning from one situation can be scaled or adapted to apply to new ones (Spanne and Jorntell, 2015). An alternative mode of representation of tactile information would be one that automatically arises out of experienced interactions through learning. Indeed, during early development, mammalians generate seemingly random movements and interactions with the environment, which would engage a wide set of sensors from large parts of the body (Shao et al., 2016) and play a crucial role for development (Forssberg et al., 1995; Petersson et al., 2003; Blumberg et al., 2013). Such interactions result in spatiotemporal patterns of skin sensor activation, that depend on, and therefore abstract, the properties of the objects we touch, the laws of physics describing the interactions that can take place (Hayward, 2011), the types of movement we make, the mechanical effects inducible in the skin and how the tactile sensors are tuned to them (Jörntell et al., 2014). The available set of sensors will respond across these conditions and their activations will have specific relationships depending on the condition. Hence, rather than viewing brain integration of tactile sensors as occurring in a pixel-by-pixel fashion, we here consider the often overlooked fact that individual neurons integrate information from several sensors. It follows that what is being learnt is likely to involve the relationships between the activations of these sensors. To learn such relationships is here hypothesized to be an important component of being able to form representations of the external world that is applicable or generalizable to novel experiences.

As tactile inputs are first processed in the cuneate nucleus, before they reach the cortex, it is likely that the basic constraints on the brain's representation of the tactile part of the physical world are formed here. *In vivo* whole cell recordings from these neurons indicate that their synaptic weights for tactile inputs are highly skewed, which indicates that they are highly learnt (Bengtsson et al., 2013). The important question why that learning occurs recently found a possible answer, when the cuneate neurons were found to code for unique combinations of the fundamental haptic input features (Jörntell et al., 2014), which tentatively correspond to the dimensions or the vector decompositions of the contact mechanics effects arising between two objects (Hayward, 2011). Here, we emulated the learning that would arise from of a set of varied sensory experiences given the biological constraints provided by the recording data we obtained on the intrinsic membrane dynamics and the synaptic inputs of the neurons of the cuneate nucleus. We find that the main effect of the cuneate learning is a utility-based decomposition of the tactile sensory space into vector components that made it possible to generalize the learning to novel tactile experiences. This is a different form of representation of sensory input data than a direct identification of sensory input patterns, which is a potential explanation for the large versatility in the identification of sensor input data in biological systems.

## Methods

All biological data was obtained under acute conditions identical to those of a previous study on the cuneate nucleus *in vivo* (Bengtsson et al., 2013). Briefly, adult cats of both sexes were initially prepared under propofol anesthesia and subsequently decerebrated to allow cuneate neuronal recordings under non-anesthetized conditions. This study was carried out in accordance with the recommendations of Malmö-Lund Animal Research Ethics Committee. All experimental procedures were approved in advance by the Malmö/Lund Animal Research Ethics Committee (permit number and approval-ID: M32-09).

This section contains four main parts. First, we describe how recordings were made from projection neurons and inhibitory interneurons of the cuneate nucleus using the *in vivo* whole cell patch clamp technique. Secondly, the recorded characteristics of the cuneate projection neurons and the inhibition from the interneurons were approximated by constructing a model of individual cuneate projection neurons (CNs) and their afferent network. Thirdly, the responses of a population of skin sensors that provided synaptic input to the CN network across a range of different real world stimuli were generated by a bionic fingertip. Fourthly, the CN synaptic learning process for skin sensor input was inferred from generic neuronal learning mechanisms *in vivo* and our estimation of intracellular calcium responses. The theoretical basis for the construction of the CN model and its learning process is also provided for each step.

### Biological data

Briefly, under initial deep propofol anesthesia, adult cats were prepared for acute *in vivo* recordings with artificial respiration, strict monitoring of blood pressure, end-expiratory carbon dioxide and temperature. Thereafter, the animals were decerebrated and the anesthesia discontinued. To monitor the level of anesthesia before decerebration, we continuously measured the blood pressure and verified the absence of withdrawal reflexes to noxious stimulation. To monitor the state of the preparation after the decerebration, we in addition made EEG recordings from intact parts of the neocortex. EEG recordings were characterized by a 1–4 Hz oscillatory activity that was periodically interrupted by large-amplitude 7–14 Hz spindle oscillations lasting for 0.5 s or more. Such EEG patterns are associated with deep stages of sleep (Niedermeyer and Da Silva, 2005). The EEG activity and the blood pressure remained stable, also on noxious stimulation, throughout the experiments. Mounting in a stereotaxic frame, drainage of cerebrospinal fluid, pneumothorax and clamping the spinal processes of a few cervical and lumbar vertebral bodies served to increase the mechanical stability of the preparation.

*In vivo* whole cell patch clamp recordings were made in the exposed cuneate nucleus. The recorded neurons were identified with respect to the location of their excitatory receptive field on the skin (Bengtsson et al., 2013). Stimulation in relation to the location of this receptive field was done using force-time controlled touch as well as by local electrical activation of skin afferents. Current injections to characterize the intrinsic membrane responses were made through the recording pipette. All intracellular signals were analyzed off-line using custom-made software. Identification of unitary IPSPs was made using a tailored template matching routine with manually constructed templates. The templates consisted of a time series of voltage ranges into which the signal had to fit to be counted. The template was adjusted to allow the detection of gradually smaller events until it started to include events that on visual inspection did not resemble the event time course of IPSPs evoked by skin stimulation (Bengtsson et al., 2013). Hence, the similarity of time course between evoked and unitary IPSPs was taken as an indication that they were derived from the same type of inhibitory synapses.

A difference with our previous analysis of EPSPs (Bengtsson et al., 2013), was that the voltage deflections of the unitary IPSPs were much smaller. Thereby, the noise of the recording system prevented the detection of IPSPs of amplitudes below a certain level. Therefore, our reported values of the median peak unitary IPSP amplitudes for each neuron are most likely overestimates. For population data, we report the mean and standard deviation of the median IPSP amplitudes recorded for at least 100 spontaneous IPSPs in each of the 15 recorded cuneate projection neurons.

### Modeling design

Based on our present biological observations, as well as previously published data on cuneate neurons (Bengtsson et al., 2013) and generic neuronal physiology and plasticity, we aimed to simulate how these parameters and processes could support learning when brought together in a functional system. Our simulation consisted of two main components,

(a) A bionic fingertip covered with silicon skin and equipped with tactile sensors that transduced local mechanical skin strain patterns into spatiotemporal patterns of sensor spike output data in response to physical interactions with external objects/surfaces. This was the counterpart of the tactile primary afferents (PAs) in biological systems. The bionic fingertip provided the important feature of a system of PA sensors where there is a consistent relationship between the activation of the different sensors across conditions or tactile experiences. As discussed in the introduction, rather than viewing brain integration of tactile sensors as occurring in a pixel-by-pixel fashion, we here consider the often overlooked fact that each cuneate nucleus neurons integrate information from several sensors. It follows that what is being learnt is likely to involve the relationships between the activations of these sensors. To learn such relationships is here hypothesized to be an important component of being able to form representations of the external world that is applicable to novel experiences.
(b) A simulated cuneate nucleus neuronal network containing a set of cuneate neurons receiving synaptic input from those PAs. Each model cuneate projection neuron (CN) was simulated individually, capturing the intrinsic responsiveness observed *in vivo* and the generic subcellular processes involved in synaptic plasticity. The accumulative effects of the synaptic plasticity during the learning process were evaluated by the changes in synaptic weights of the PA inputs in each CN.

In the account that follows, we present the design of the simulation starting with the low level neuronal properties, the dynamic model describing the intrinsic responsiveness of the cuneate neurons, synaptic weight initialization, the sensory input data and the bionic fingertip used to generate it, the learning process and the evaluation of the end effect of the learning process. The design and performance of the bionic fingertip has already been reported in previous papers, therefore we merely present the specific adaptations made for the present simulation. The modeling software is available on: https://figshare.com/projects/Artificial_Cuneate_Neuron_Model/34886.

### Network connectivity

**Figure 1:**
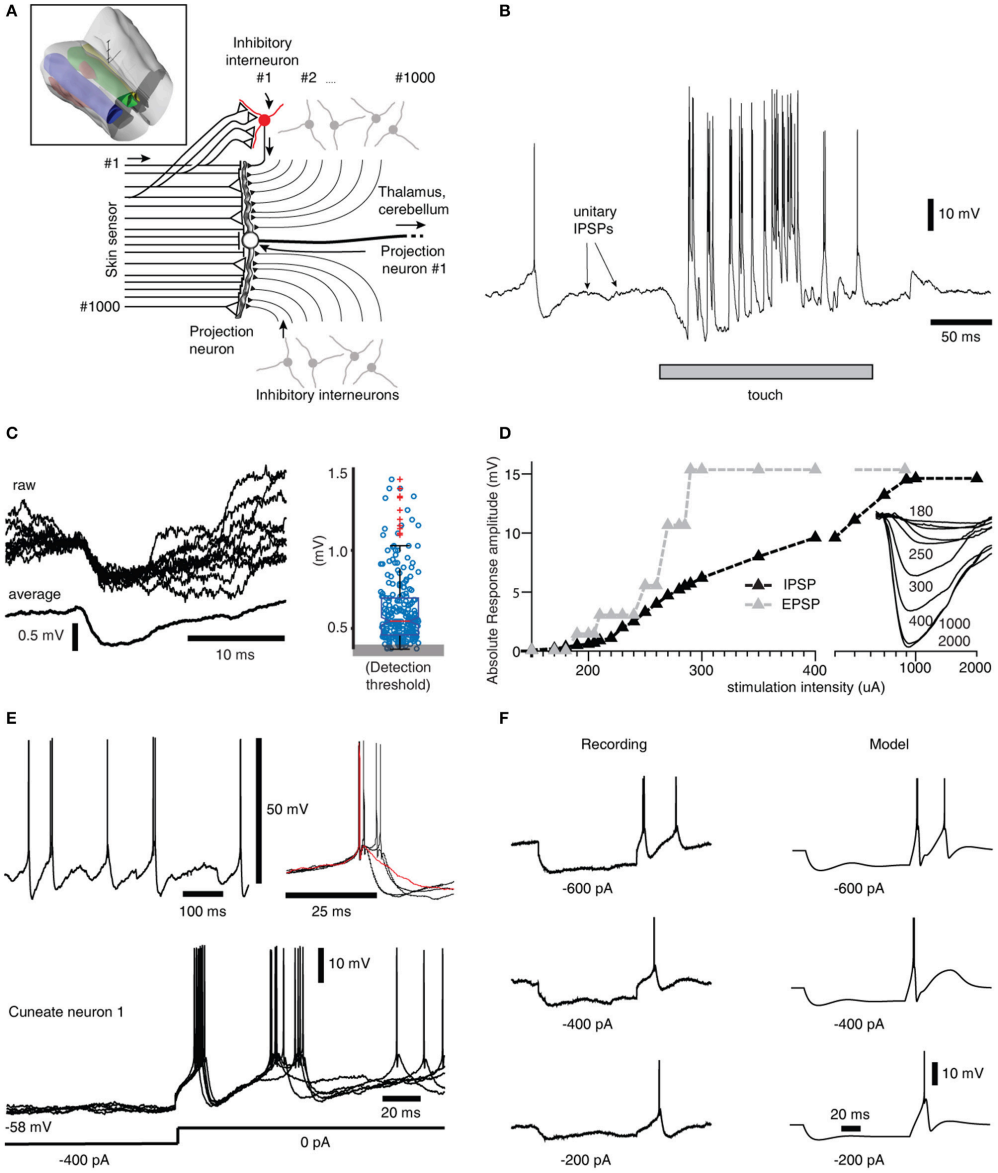
Inhibitory synaptic inputs and intrinsic responses of cuneate neurons *in vivo*. **(A)** Schematic of cuneate nucleus neuronal circuitry. Lines from the left symbolize axons originating from skin sensors. Their synaptic weights are indicated by the size of the triangles or as a stop ending for near-zero weights. Inhibitory interneurons (red for highlighted neuron, the others are gray) send axons that make inhibitory synapses on the projection neuron (black triangles). In the middle is a cuneate projection neuron, which sends its axon to the thalamus or the cerebellum. Inset: 3D illustration of the lower brainstem and the location of the cuneate nucleus (green). 3D scale bar indicate distances (1 mm per division). Yellow is gracile nucleus, small red volume is external cuneate nucleus. **(B)** Response of a projection neuron *in vivo* to a light skin touch within its receptive area. Arrows indicate putative spontaneous IPSPs, which are so small they are barely visible at this magnification of the voltage trace. **(C)** At a different magnification, examples of spontaneous IPSPs from one cuneate projection neuron are superimposed and averaged to the left. The peak amplitudes of 500 consecutive spontaneous IPSPs are shown in the box plot at right. **(D)** The gradual recruitment of summated, or compound, IPSPs with increased electrical stimulation intensity to a skin area adjacent to the excitatory receptive skin area of a sample projection neuron (black curve). Gray curve illustrates corresponding recruitment of unitary EPSPs (Bengtsson et al., 2013). Inset traces, average evoked IPSPs (averages of 20-50 repetitions) at different stimulation intensities. **(E)** Spontaneous activity of a projection neuron with zoomed-in, superimposed spikes to the right. Red trace indicates a case of a spontaneous single spike event, whereas most spontaneous firing occurred in doublets with an associated difference in voltage trajectory after the spike. Bottom, examples of rebound responses elicited by release from hyperpolarizing current injections (−400 pA for 200 ms). **(F)** Comparison between the rebound responses of a cuneate neuron *in vivo* and the CN model.

**Figure 2:**
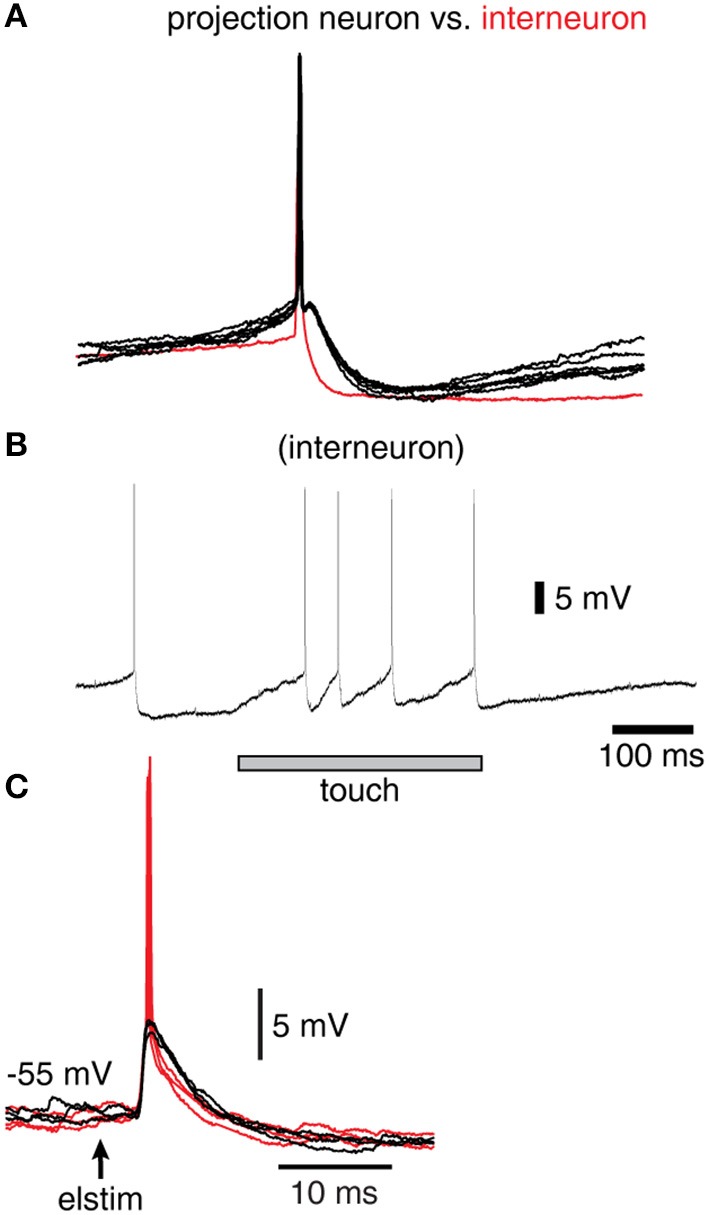
Spike responses in a sample interneuron of the cuneate nucleus. **(A)** Recordings were identified as projection neurons (black traces) and interneurons (red trace) based on their characteristic spike shapes. **(B)** Example response of an interneuron evoked by a brief touch to the skin. **(C)** Response of a sample interneuron to electrical skin stimulation. Black traces indicate evoked EPSPs that did not elicit a spike whereas red traces indicate those that did.

**Figure 3:**
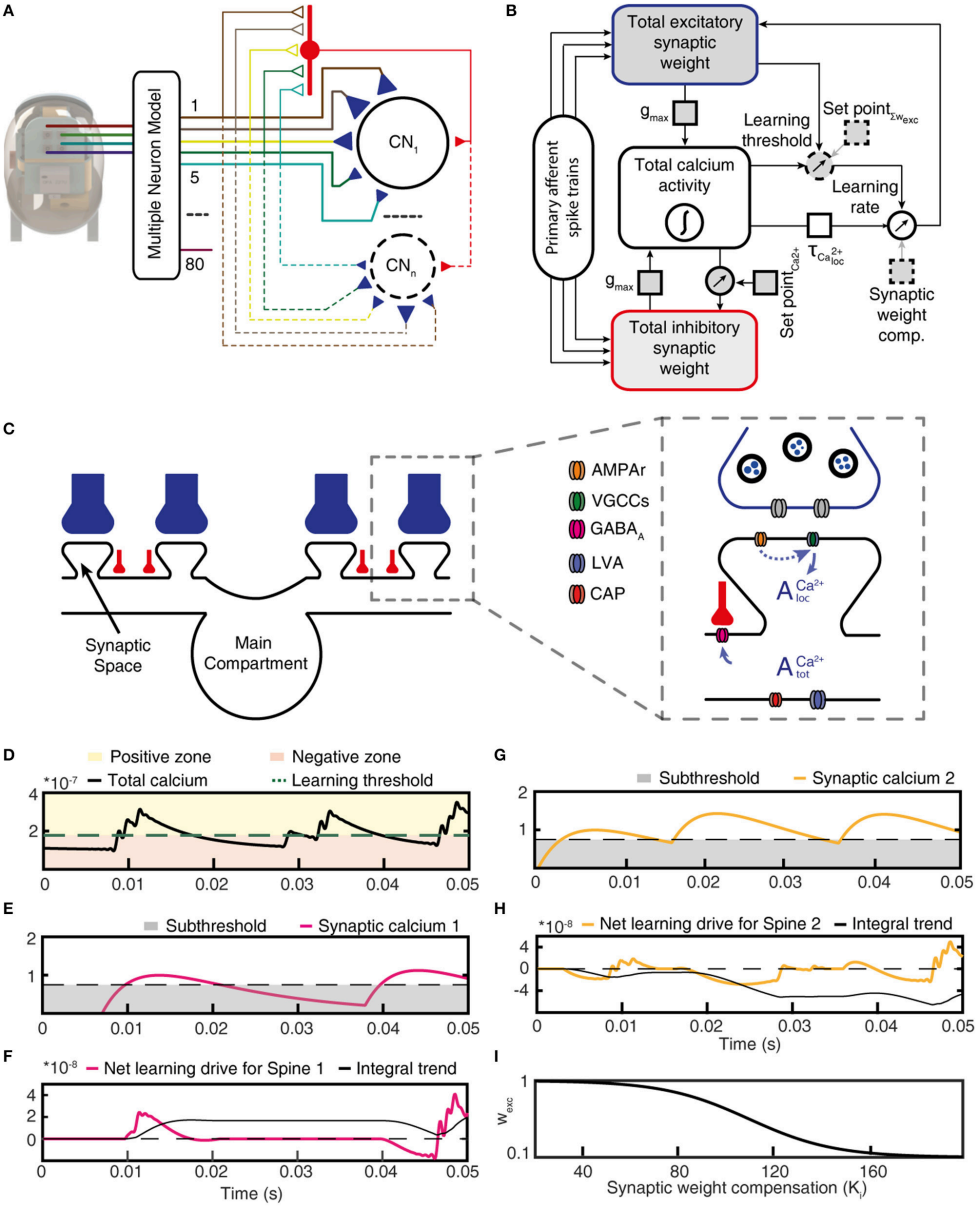
Functional structure of the CN model. **(A)** Structure of the CN network model. Colored lines indicate PA connections between the physical sensors of the bionic fingertip and the neurons of the model, blue triangles indicate variable weight synapses. Only cuneate projection neurons (CN1–CNn) were simulated individually, where the number identifies a specific initial synaptic weight configuration (see Figure 5B). PA inputs were also provided to the CNs as a lump inhibitory synaptic input via an interneuron (red). **(B)** Graphic representation of the functional structure of the CN model. Components indicated with a dashed outline were the only elements with fixed parameters, whereas parameters with solid outlines were adjusted in the later part of this paper to simulate CNs with different intrinsic properties. **(C)** Subcellular factors involved in the synaptic plasticity of the CN, with variable weight PA synapses (blue) and inhibitory synapses (red). The CN neuron is divided into a main compartment, with reactive LVA and CAP conductances and synaptic spaces containing VGCCs (Higley and Sabatini, 2012) and a variable number of AMPAr:s. AMPAr, excitatory glutamate receptors; VGCCs, voltage gated calcium channels; GABAa, inhibitory synaptic receptors; LVA, low–threshold voltage activated calcium channels; CAP, calcium-dependent potassium channels. **(D)** The calcium activity of the main compartment varied over time due to synaptic input and the responses they elicited via the intermediate dynamics model of the CN. The level of the calcium activity, in relationship to the learning threshold, defined when the cell was in the positive zone (i.e., potentiation mode) or in the negative zone (depression mode). **(E)** The local calcium activity threshold used to define the eligibility for plasticity for each PA synapse. **(F)** The net learning drive for the PA synapse varied depending on the temporal correlation between the zero-offset main compartment calcium activity and the local, or synapse space, calcium activity. **(G)** The activity of another PA synapse under the same stimulus presentation and; **(H)** its net learning drive. **(I)** The synaptic weight compensation constant that was multiplied with the integral net learning drive to calculate the final weight change per stimulus presentation. For **(D,E,G)**, note that the y axes indicate the relative magnitude of calcium signals for each compartment and not an actual estimation of the calcium concentration.

Based on biological data of cuneate neurons (Bengtsson et al., 2013; Figures 1, 2), our network model (Figure 3A) was comprised of sensory afferents projecting as excitatory synapses on each individual model CN. In addition to the excitatory synapses, and based on our biological observations (Figure 1), the sensory afferents also drove local inhibitory interneurons that provided inhibitory synapses to the CNs (Figure 3A). The model CN had equal numbers of excitatory and inhibitory synapses. The synaptic weights of these synapses were given initial starting weights (or seed weights, see below) that were gradually learnt on a trial-by-trial basis.

The simulated network model had 80 sensory input channels (PAs) that innervated each individual CN. This number of afferents was less than biological estimates, which suggest in the order of 1,000's of PAs per CN (Bengtsson et al., 2013). However, the lower number of simulated afferents is still realistic because out of these 1,000 synapses, many are likely to represent anatomically present synaptic inputs from PA axons mediating input from several different fingers, which in the adult animal mostly provide “silent” synaptic inputs (Weinberg et al., 1990; Bengtsson et al., 2013). In contrast, our inputs were generated from the tip of one finger alone. Synaptic inhibition was simulated as being provided by 80 independent interneurons that were each directly activated by one out of the 80 PA afferents available in our simulated system. Because all unitary inhibitory synaptic inputs were found to be of comparable, low weights in the biological cuneate neuron (Figure 1), inhibitory synapses were here simplified to one lump inhibitory synapse per CN (total inhibitory synaptic weight, Figure 3). Hence, interneurons were not explicitly simulated, but instead the spiking activity of each PA afferent was fed directly to an inhibitory synapse on the CN.

### Neuron model

The model cuneate projection neuron (CN) was implemented as a conductance based Exponential Integrate and Fire (EIF) model (Fourcaud-Trocmé et al., 2003), which can recreate the fast dynamics (~1 ms timescale) of neuronal spike generation. In addition to the basic EIF model, voltage sensitive calcium channels and calcium dependent potassium channels were also modeled in order to recreate intermediate cuneate neuron dynamics (~10 ms timescale) observed *in vivo* (Figures 1E,F). The complete dynamics of the CN membrane potential are given by:

(1) $$\begin{array}{r} C_{m}\frac{dV_{m}}{dt}=\;I_{L}+I_{spike}+I_{ion}+I_{ext}+I_{syn} \end{array}$$

where *C*_*m*_ is the membrane capacitance, $I_{L}=\;{-\bar{g}}_{L}\left(V_{m}-E_{L}\right)$is the leak current, *I*_*spike*_ is the spike currents (fast dynamics), *I*_*ion*_ is the ion channel currents (intermediate dynamics), *I*_*syn*_ is the synaptic input currents and *I*_*ext*_ denotes external injected currents that were used to evaluate the intrinsic CN neuron responsiveness to current step commands (Figure 1F). The model values for these and other parameters are given in Table 2. The membrane resistance and time constant/capacitance were within the range of values recorded *in vivo* (Bengtsson et al., 2013), whereas other intrinsic parameter values were chosen through an optimization process (see below).

### Neuron model-fast dynamics

The spike current (*I*_*spike*_) is generated using a basic EIF model (Equation 2) to achieve the fast dynamics and recreate the initiation of the action potential (Ostojic and Brunel, 2011).

(2) $$\begin{array}{r} I_{spike}=\;{\bar{g}}_{L}\;\Delta_{T}\;\exp\left(\frac{V_{m}-V_{t}}{\Delta_{T}}\right) \end{array}$$

As the depolarization reaches a threshold speed, an action potential is said to be generated, and the membrane potential is immediately reset by a fast afterhyperpolarization (AHP) to the membrane potential at which the spike was initiated, i.e., mimicking the fast spike AHP of the recorded cuneate projection neurons (see Figure 2A).

### Neuron model-intermediate dynamics

The intermediate dynamics include currents from additional ion channels (*I*_*ion*_, Equation 1) that are not directly involved in forming action potentials, but have more of a modulating role in episodes leading up to the generation of action potential and the episodes between action potentials when the synaptic input activity is high. The intermediate dynamics of the model were optimized to mimic the reactive conductances that could generate the types of responses to current injections we recorded in the cuneate neurons *in vivo* (Figures 1E,F). Such responses, i.e., post-inhibitory rebound responses and a tendency to generate bursts of action potential firing, have been observed in other neuron types (Llinás and Jahnsen, 1982; Huguenard, 1996; Molineux et al., 2008) and has at least partly been attributed to by *low-threshold voltage gated calcium channels* (LVA) and *calcium-activated potassium channels* (CAP). Hence, *I*_*ion*_ can be divided according to:

(3) $$\begin{array}{r} I_{ion}=\;I_{Ca}+\;I_{K} \end{array}$$

where *I*_*Ca*_ is the current through LVA channels or equivalent channels and *I*_*K*_ the current through the CAP channels. These are modeled as two separate pools of ion channels (Saarinen et al., 2008) according to:

$$\begin{array}{r} I_{Ca}=-{\bar{g}}_{Ca}x_{Ca,a}^{3}x_{Ca,i}\left(V_{m}-E_{Ca}\right) \end{array}$$

(4) $$\begin{array}{r} I_{K}=-\;{\bar{g}}_{K}x_{K_{Ca}}^{4}x_{K_{Vm}}^{4}\left(V_{m}-E_{K}\right) \end{array}$$

where ${\bar{g}}_{Ca}$ and ${\bar{g}}_{K}$ are the maximum conductances of the respective channels, *E*_*Ca*_ and *E*_*K*_ are the reversal potentials of the respective ions, and *x*_*K*_*Ca*__, *x*_*K*_*Vm*__, *x*_*Ca, a*_, *x*_*Ca, i*_ are the activity states of the channels (Saarinen et al., 2008).

The activation states of the LVA channels were modeled using the following differential equations:

$$\begin{array}{r} \frac{d\left(x_{C_{a,a}}\right)}{dt}=\left({\bar{x}}_{C_{a,a}}\left(V_{m}\right)-x_{C_{a,a}}\right)/\tau_{C_{a,a}} \end{array}$$

(5) $$\begin{array}{r} \frac{d\left(x_{C_{a,i}}\right)}{dt}=\left({\bar{x}}_{C_{a,i}}\left(V_{m}\right)-x_{C_{a,i}}\right)/\tau_{C_{a,i}} \end{array}$$

where tau is the time constant at which the states move toward the voltage dependent equilibrium described by ${\bar{x}}_{Ca}\left(V_{m}\right)$. These equilibrium functions are two parameters sigmoid of the form:

$$\begin{array}{r} {\bar{x}}_{Ca,a}\left(V_{m}\right)={\left(\text{1}+\exp\frac{p_{Ca,a,1}-V_{m}}{p_{Ca,a,2}}\right)}^{-1} \end{array}$$

(6) $$\begin{array}{r} {\bar{x}}_{Ca,i}\left(V_{m}\right)=\text{1}-{\left(\text{1}+\exp\frac{p_{Ca,i,1}-V_{m}}{p_{Ca,i,2}}\right)}^{-1} \end{array}$$

Since the calcium sensitive subunits of the CAP channels are located on the inner surface of the cell membrane, the intracellular concentration of calcium ([*Ca*^2+^]) is modeled for small volumes rather than as an overall concentration within the cell. The calcium concentration within the cell will change both due to ion channels through which calcium ions enter to the inside of the membrane, and diffusion of ions into the remaining intracellular volume. From this line of reasoning, and in accordance with Saarinen et al. (2008), Equation 7 is constructed

$$\begin{array}{rcl} \frac{d\left(\left[Ca^{2+}\right]\right)}{dt} & = & B_{Ca}\bar{g}C_{a}x_{C_{a,a}}^{3}x_{C_{a,i}}\left(V_{m}-E_{Ca}\right) \end{array}$$

(7) $$\begin{array}{r} +\;\left({\left[Ca^{2+}\right]}_{rest}-\left[Ca^{2+}\right]\right)/\tau_{\left[Ca^{2+}\right]} \end{array}$$

The activation states of the CAP channels were modeled using the following differential equations:

$$\begin{array}{r} \frac{d\left(x_{K_{Ca}}\right)}{dt}=\left({\bar{x}}_{K_{Ca}}\left(\left[Ca^{2+}\right]\right)-x_{K_{Ca}}\right)/\tau_{K_{Ca}} \end{array}$$

(8) $$\frac{d(x_{K_{Vm}})}{dt}=({\bar{x}}_{K_{Vm}}(V_{m})-x_{K_{V_{m}}})/\tau_{K_{Vm}}$$

where the two time constants (τ_*K*_*Ca*__ and τ_*K*_*Vm*__) indicate the times at which the states move toward the voltage dependent equilibrium described by ${\bar{x}}_{K_{Vm}}$ and the calcium dependent equilibrium described by ${\bar{x}}_{K_{Ca}}$. These equilibrium functions are two parameters sigmoid of the form:

$$\begin{array}{r} {\bar{x}}_{K_{Vm}}\left(V_{m}\right)={\left(\text{1}+\exp\frac{p_{K_{Vm},1}-V_{m}}{p_{K_{Vm},2}}\right)}^{-1} \end{array}$$

(9) $$\begin{array}{r} {\bar{x}}_{K_{Ca}}\left(\left[Ca^{2+}\right]\right)=\text{1}-{\left(\text{1}+\exp\frac{p_{K_{Ca},1}-\left[Ca^{2+}\right]}{p_{K_{Ca},2}}\right)}^{-1} \end{array}$$

The values for these parameters were chosen through an optimization procedure (see below) and are indicated in Table 2. Note that we observed a range of variance between cuneate neurons *in vivo* in terms of their intermediate dynamics. Our aim was to provide a simple model that at the same time could capture the main principles of the rebound and burst responses that we could demonstrate in our recorded cuneate neurons. Therefore, we do not expect that our parameter values have a direct correspondence with biophysical measures, and we do not expect to precisely capture the properties of the intermediate dynamic response of any single neuron (which likely would have required a larger set of parameters).

### Neuron model-synaptic inputs

The synaptic current (*I*_*syn*_) through the cell membrane is the summated synaptic currents of the activated synapses. Each individual synapse (*i*) is activated by a primary afferent spike generated by a single sensor of the bionic fingertip. Once activated, this spike gives rise to a stereotyped time course of conductance injection at the synapse (*I*_*syn*_) which is described by

$$\begin{array}{r} I_{syn}=g_{\max}\underset{i}{\sum }w_{exc,i}\exp\left(-\tau \left(t-t^{{}^{*}}\right)\right)\left(E_{rev,exc}-V_{m}\right) \end{array}$$

(10) $$\begin{array}{r} +g_{\max}w_{inh}\underset{i}{\sum }\exp\left(-\tau \left(t-t^{{}^{*}}\right)\right)\left(E_{rev,inh}-V_{m}\right) \end{array}$$

where *E*_*rev*_ is the reversal potential of the type of synapse (*E*_*rev, exc*_ or *E*_*rev, inh*_ depending on whether the synapse is excitatory or inhibitory, see Table 2), *V*_*m*_ is the membrane potential and t^*^ is the time of activation of the synapse. Each spike in each sensory afferent was converted into a synaptic conductance in the simulated neuron. For each synapse, the peak amplitude of the synaptic response was determined by the product of their individual weight (*w*_*exc*_ or *w*_*inh*_) and the overall *maximum synaptic conductance constant* (*g*_max_, see Table 1). Through *g*_max_ the relative leak conductance (i.e., the ratio of the synaptic and the leak conductances) could be adjusted to simulate cuneate neurons with different sizes. The time constant of the decay of the synaptic membrane potential responses, τ, was 6.4 ms for both excitatory and inhibitory synapses, in accordance with the time courses recorded in the cuneate nucleus neurons *in vivo* (Bengtsson et al., 2013; Figure 1). Note that as all the PA synapses of our system stayed well below 200 Hz of firing activity, we did not simulate any rate adaptation of the PA synapses as such adaptation *in vivo* primarily occurs at intervals shorter than 5 ms (Jörntell and Ekerot, 2006).

**Table 1:** Specification of the intrinsic CN parameter values used.

**Parameters**	**Initial value/range**
Maximum synaptic conductance constant (*g*_max_)	23e-9 S, 9e-9 S, **23e-8 S**, 9e-7 S, 23e-7 S
Calcium activity set point ($SetPoint_{Ca^{2+}}$)	**20 Hz**, 40 Hz, 60 Hz, 80 Hz, 100 Hz
Synaptic local Ca^2+^ time constant ($\tau_{Ca_{loc}^{2+}}$)	[τ_*d*_, τ_*r*_, τ_*m*_] = 50%, **100%**, 150%, 200%, 250%
Seed weight distribution spread (SW Spread)	**0%**, −20%, −40%, −60%, −80%

*The values of the basic CN configuration, or default values, are indicated in bold*.

### Optimization of the neuronal calcium dynamic model against measured data

The complete model was optimized during three steps. During each simulation, the model was fed with six 100 ms current steps with amplitudes of 100, 200, 300, 400, 600, and 800 pA. The results from the six trials were then optimized against intracellular recordings where the cuneate neurons were fed with the same currents *in vivo* (Figure 1F).

The first step is to manually choose suitable initial parameters, using both previously known values to some of the parameters, and estimating others using trial and error simulations. The second step is to use the Nelder-Mead algorithm, but with an objective function where the simulated traces is compared to the measured traces. The third step also uses the Nelder-Mead algorithm, but with an objective function that measure the discrepancy between the action-potential timing in the simulated trace and the measured trace. As there is no guarantee that the simulated trace contains the same number of action potentials as the measured trace, discontinuities appear when the number of action potentials in the simulated trace change. The use of the Nelder–Mead method is motivated by that this is a commonly applied numerical method used to find the minimum or maximum of an objective function in a multidimensional space, in particular for nonlinear optimization problems. Equation 11 contains the complete objective function:

(11) $$e=\;\{\begin{array}{c} e_{t}\omega_{s}\sum_{T,\hat{T}\in T}{\left(|\hat{T}|-|T\;\right)}^{2}\;\begin{array}{c} \exists T,\hat{T}\in T\;|\hat{T}|\neq \end{array}|T|\\ \;e_{t}\;\begin{array}{c} \forall T,\hat{T}\in T\;|\hat{T}|= \end{array}|T| \end{array}$$

where *e* is the objective function value, ω_*s*_ a weight used to punish any discrepancy in the number of action potentials, and *e*_*t*_ the total time error between simulated and measured action potentials:

(12) $$e_{t}=\underset{T,\hat{T}\in T}{\sum^{\text{​}}}\{\begin{array}{cc} \underset{t\in T}{\sum^{\text{​}}}\underset{{}_{\hat{t}\in \hat{T}}}{\min}|t-\hat{t}{|}^{2} & \left|T|\geq |\hat{T}\right|\\ \underset{\hat{t}\in \hat{T}}{\sum^{\text{​}}}\underset{t\in T}{\min}|t-\hat{t}{|}^{2} & \left|T|<|\hat{T}\right| \end{array}$$

where 𝕋 is the set of all pairs of $T,\hat{T},$ where *T* and $\hat{T}$ are the sets of all measured and simulated action potentials, respectively, during a single current step. The list of all optimized parameters used in the model is shown in Table 2.

**Table 2:** Neuron model parameters.

**Parameter**	**Symbol**	**Value**
Membrane capacitance	*C*_*m*_	4.270e-11 F
Leak conductance	*g*_*L*_	8.100e-09 S
Leak reverse potential	*E*_*L*_	−62.309 mV
Width of the spike (EIF model)	Δ_*t*_	1.3063
Spike threshold (EIF model)	*V*_*t*_	−57.129 mV
Maximum potassium conductance	*g*_*K*_	2.022e-08 S
EPSP reversal potential	*E*_*rev, exc*_	0 mV
IPSP reversal potential	*E*_*rev, inh*_	−80 mV
Potassium reversal potential	*E*_*K*_	−104.514 mV
Maximum calcium conductance	*g*_*Ca*_	2.082e-08 S
Calcium reversal potential	*E*_*Ca*_	121.436 mV
Conversion factor between calcium current and concentration	*B*_*Ca*_	3.374e-15
Calcium concentration at rest (equilibrium)	$Ca_{rest}^{2+}$	1.010e-07
Time constant of the calcium concentration leak	$\tau_{Ca^{2+}}$	0.0063
Time constant of the calcium activation state	τ_*Ca, a*_	2.722e-04
Time constant of the calcium inactivation state	τ_*Ca, i*_	0.0207
Time constant of the potassium calcium dependent activation state	τ_*K*_*ca*__	0.0013
Time constant of the potassium voltage gated activation state	τ_*K*__*V*__*m*___	0.0011
Constant for sigmoid function of intermediate dynamic model	*p*_*Ca, a*, 1_	−60.8369 mV
Constant for sigmoid function of intermediate dynamic model	*p*_*Ca, a*, 2_	6.3419 mV^−1^
Constant for sigmoid function of intermediate dynamic model	*p*_*Ca, i*, 1_	−68.0100 mV
Constant for sigmoid function of intermediate dynamic model	*p*_*Ca, i*, 2_	1.3008 mV^−1^
Constant for sigmoid function of intermediate dynamic model	*p*_*K*_*Vm*_, 1_	−64.0785 mV
Constant for sigmoid function of intermediate dynamic model	*p*_*K*_*Vm*_, 2_	0.7833 mV^−1^
Constant for sigmoid function of intermediate dynamic model	*p*_*K*_*Ca*_, 1_	2.2166e-07 mV
Constant for sigmoid function of intermediate dynamic model	*p*_*K*_*Ca*_, 2_	4.7923e-08 mV^−1^

### Subsynaptic local calcium activity

In the learning process, excitatory synaptic weight learning was driven by the calcium activity in the main compartment of the cuneate neuron (i.e., as calculated by the calcium dynamic model above) in combination with the calcium activity in the individual synapses. An essential component of this combination is the intensity of activation of the individual synapses. According to the learning rule that we used (see below), a synapse that fires at high frequency with a high degree of correlation with the main compartment total calcium activity ($A_{tot}^{Ca^{2+}}=k\left[Ca^{2+}\right]$, where k is an arbitrary constant that is here assumed to be 1), will be “rewarded” as due to the strong correlation with the learning signal $A_{tot}^{Ca^{2+}}$. Conversely, strong firing in a synapse in relation to low or zero $A_{tot}^{Ca^{2+}}$ will be “punished” (i.e., similar to the classical BCM rule for Hebbian plasticity) (Bienenstock et al., 1982). Therefore, the *local calcium time constants* ($\tau_{Ca_{loc}^{2+}}$, Table 1), defining the temporal properties of the calcium signal in the local space underneath each individual synapse, play a major role in the learning process (the local postsynaptic calcium activity can be considered an analogy with the calcium activity in a local dendritic spine; Koester and Sakmann, 1998; Tigaret et al., 2016). The learning rule critically depends on this time constant. For instance, if $\tau_{Ca_{loc}^{2+}}$ time constants are too high, the rewarding effects on synapses that have a high degree of correlation with the $A_{loc}^{Ca^{2+}}$ will be lost. However, as there is no data on the relevant time constants in the cuneate neurons *in vivo*, we had to make assumptions of the values of this time constant. In order to avoid pitfalls in relation to this assumption, we studied a range of time constants for $A_{loc}^{Ca^{2+}}$ (Table 1) during the CN learning process.

For each synapse, each input spike at time t^*^ contributes to the subsynaptic spine calcium concentration, the time course of which is given by the kernel (Mazzoni et al., 2008) of Equation 13,

$$\begin{array}{rcl} \alpha & = & \frac{\tau_{1}}{\tau_{d}-\tau_{r}} \end{array}$$

(13) $$\begin{array}{rcl} \Delta A_{loc}^{Ca^{2+}}\left(t\right) & = & \;\alpha *\left[\exp\left(-\frac{t-\tau_{l}-t^{{}^{*}}}{\tau_{d}}\right)\;-\;exp\left(-\frac{t-\tau_{l}-t^{{}^{*}}}{\tau_{r}}\right)\right] \end{array}$$

In its basic configuration, the parameters describing the relative local calcium concentration (or activity), are the decay time τ_*d*_ = 12.5 ms and the rise time τ_*r*_ = 4 ms multiplied with a constant τ_1_ = 21 ms (which is a constant to calculate the ratio) for $\tau_{Ca_{loc}^{2+}}$ = 100% (Table 1). τ_*l*_ is the latency time which is zero in our case. The initial values chosen were derived from our assumption that the time course of the slow afterhyperpolarization of the cuneate neuron spike (Figure 1B), which is known to reflect the activation of calcium-dependent potassium channels, matched the time course of the calcium concentration induced in the synapse. This resulted in a somewhat faster but still comparable time course of spine calcium than reported for single spines *in vitro* (Tigaret et al., 2016), but the rise time of our subsynaptic calcium signal was slower than in previous simulations of the properties of calcium-dependent learning (Graupner and Brunel, 2012). As the temporal properties of this calcium signal clearly were assumptions with large uncertainty, we tested a wide range of different values for these parameters (Table 1). In order to achieve supralinearity in the local calcium activity (Wang et al., 2000), we used an approximative approach of subtracting an offset from the local calcium signal (an offset corresponding to 75% of the peak activity of the single pulse activation was subtracted) and this was the value of the local calcium activity ($A_{loc}^{Ca^{2+}}$). With this approach, repetitive activation of the same synapse resulted in a supralinear increase in the intensity of the local calcium activity depending on the frequency of the PA afferent activity for that synapse.

### Synaptic weights

The synaptic weight in our model was a gain factor ranging between 0.001 and 1, where 1 signified the maximum weight of a synapse. As there is no information on perinatal synaptic weights for the CNs, we needed to make assumptions. The first assumption was that synapses have different initial synaptic weights, or seed weights. The second assumption was that all synapses had initial non-zero up to medium strong synaptic seed weights. In our model, the distribution of synaptic weights across the primary afferent synapses of the CN model, or the *initial excitatory synaptic weights* (*w*_*init, exc*_), were normal distributions ranging between 0.001 and 0.5 across the 80 primary afferent inputs. We used 5 different pseudo-randomized initial weight distributions, referred to as “Seed weights 1–5.” Pseudo-randomization here implies that the distribution is randomized but that the same distribution is kept constant across different learning process runs. This had the advantage that the effects of specific intrinsic CN configurations (see “Variations of initial intrinsic CN parameters” below) could be tested for the same initial weight distributions (Figure 12).

Synaptic inhibition was simulated as being provided by 80 independent interneurons that were each directly activated by one out of the 80 PA afferents available in our simulated system. Each PA synapse had the same weight on its targeted inhibitory interneuron. The collective, or total, *inhibitory synaptic weight* (*w*_*init, inh*_) was initially set to 0.125 evenly distributed across all the inhibitory synapses between the interneurons and the CN neuron they contacted, meaning that each PA synapse provided equally weighted inhibition to the CN neuron (as suggested by our biological data accounted for in the Results).

### Sensory inputs

Our aim was to simulate the learning process in the cuneate neurons driven by simulated sensory activation of the PAs. The main idea was to simulate a varied set of interactions between the skin and the physical world, and let the resulting spatiotemporal patterns of skin sensor activation determine the outcome of the learning process in the cuneate neurons. Rather than designing an arbitrary set of spatiotemporal patterns of skin sensor activations, we wanted to use a physical model of a fingertip to generate the spatiotemporal spike patterns of PA input that the cuneate neurons of our model (CNs) learned from. This is because across the different kinds of interactions that the skin may experience, there may be relationships between the skin sensors that are not easily calculated across all conceivable conditions/interactions.

### Bionic fingertip

**Figure 4:**
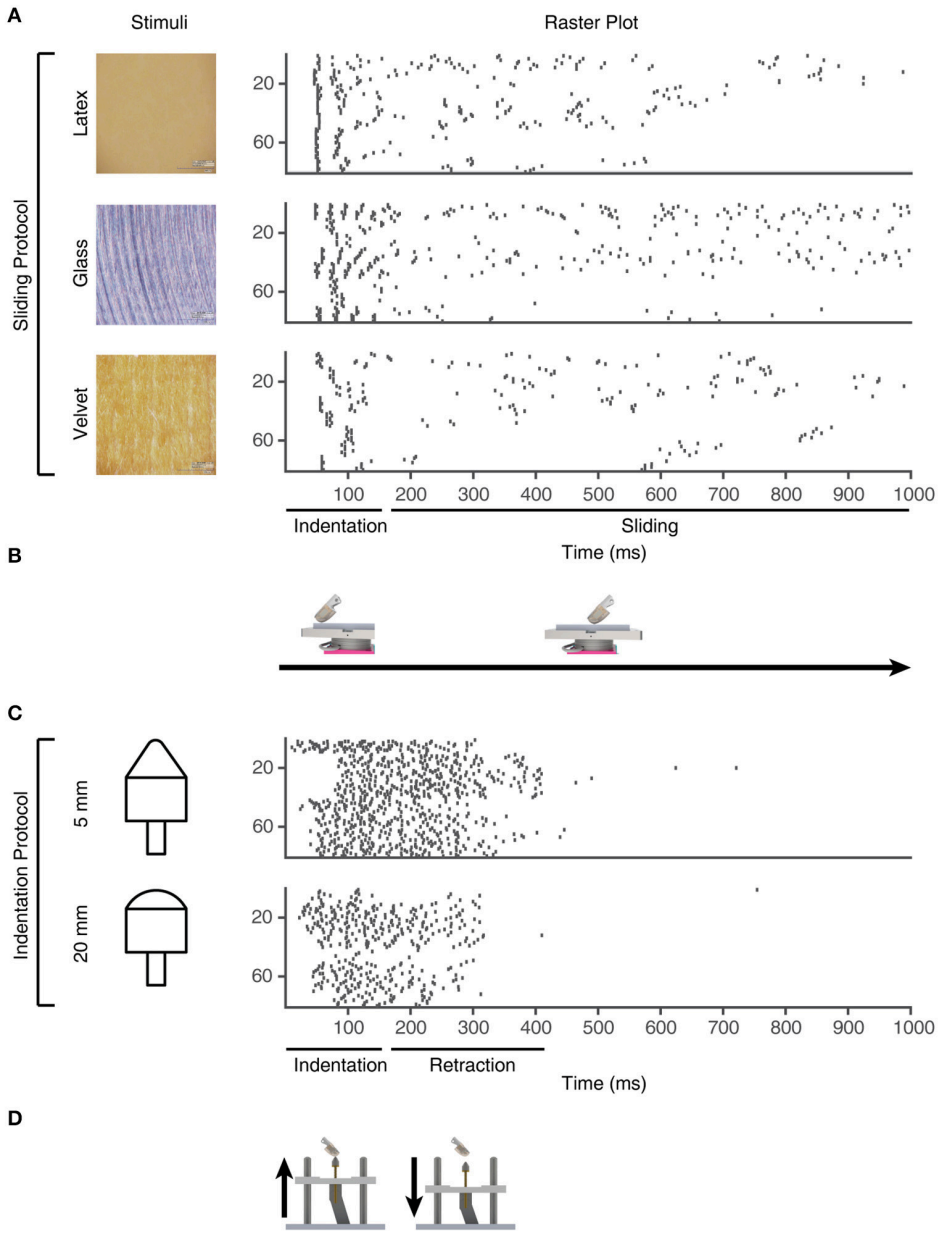
Training stimuli. Physical stimuli and corresponding sensor spiking responses in PA sensory inputs #1-80 for the training stimuli used in the learning process. **(A)** Three of the physical stimulus conditions consisted in the artificial fingertip first indenting and subsequently sliding across surfaces with different textures, as shown in **(B)**. **(C)** The two indentation stimuli, where the artificial fingertip was dynamically indented against two different shapes, as shown in **(D)**.

To generate the sensor patterns, we used a set of touch protocols based on different stimulus conditions (Table S1). The sensory fingertip comprises a 2 × 2 array of Micro Electro Mechanical System (MEMS) based bio-inspired tactile sensors (Oddo et al., 2011) to generate spatio-temporal sensory input patterns (Figure S2). Each individual sensor comprises a four transducing piezoresistors mesh (totaling 16 sensory channels for each fingertip), arranged in cross-shaped fashion able to generate precise response magnitudes for both normal and tangential forces, applied across the surface of the sensor (Beccai et al., 2005; Oddo et al., 2007). However, only four of the 16 sensor channels were sufficiently dynamically sensitive to the range of forces arising for the stimuli used to be used in the present experiment. We created 80 PA sensory input channels from these four sensors' analog data (Figure 4) by multiplexing them with multiple neuron models and signal processing as explained below. Each of these 80 PA sensor spike output patterns were considered a unique PA signal provided as synaptic input to the CNs, where sensor#1 was used to create PA input #1-20, sensor #2 to create PA input #21-40 and so on.

The normalized analog output of the tactile sensor is fed as virtual current input to a fine-tuned Izhikevich neuron models (Izhikevich, 2003) in-order to achieve the spatiotemporal spike output (Figure S2) as described previously (Oddo et al., 2016; Rongala et al., 2017). The Izhikevich model was chosen in order to reproduce the adaptation dynamics, that is a characteristic of mechanoreceptors (Johansson and Flanagan, 2009). Per the Izhikevich model, the membrane potential v and the adaptation variable u were updated via the following nonlinear differential equations discretized using Euler's method:

(14) $$\begin{array}{r} \overset{{}^{\circ}}{v}=Av^{2}+\;Bv+C-u+\;\frac{I_{input}}{C_{m}} \end{array}$$

$$\begin{array}{r} \overset{{}^{\circ}}{u}=a\left(bv-u\right) \end{array}$$

When the membrane potential reached the spike depolarization threshold of 30 mV, one spike was produced followed by a reset:

(15) $$if\;v\;\geq 30\;mV,\;then\;\{\begin{array}{c} v\leftarrow c\\ u\leftarrow u+d \end{array}$$

The *A, B, C* and the spiking threshold are the standard ones of the Izhikevich artificial neuron model, whereas the parameters *a, b, c* and *d* were selected (Table S2) to allow a regular spiking behavior.

From the existing 16 sensory channels, we consider 4 active channels (2, 5, 12, and 15) (Figure S2). We further derivated the analog sensor data to mimic both Merkel Slowly Adapting (SA) type I mechanoreceptors, sensitive to the sustained level of mechanical interactions, and Meissner Fast Adapting (FA) type I mechanoreceptors, mostly sensitive to dynamic changes (Vallbo and Johansson, 1984; Abraira and Ginty, 2013). For each physical stimulus, the stimulation was repeated five times and the corresponding consecutive five analog signals were considered as the response of a unique sensor. As we had four physical sensors, this procedure thus generated 20 analog unique sensory signals per physical stimulus. In addition, each analog sensor signal was derivated to obtain a total of 40 different analog sensory signals (Figure 4). Moreover, by implementing the Izhikevich neuron model (Izhikevich, 2003) with two different settings, we obtained a total of 80 different PA spike output patterns for each physical stimulus.

For the learning process, we used the sensor outputs obtained using five different physical stimuli (Figure 4), and in addition the PA spike output responses to five other physical stimuli were provided as “non-training” stimuli (see Results text).

### Learning protocol

**Figure 5:**
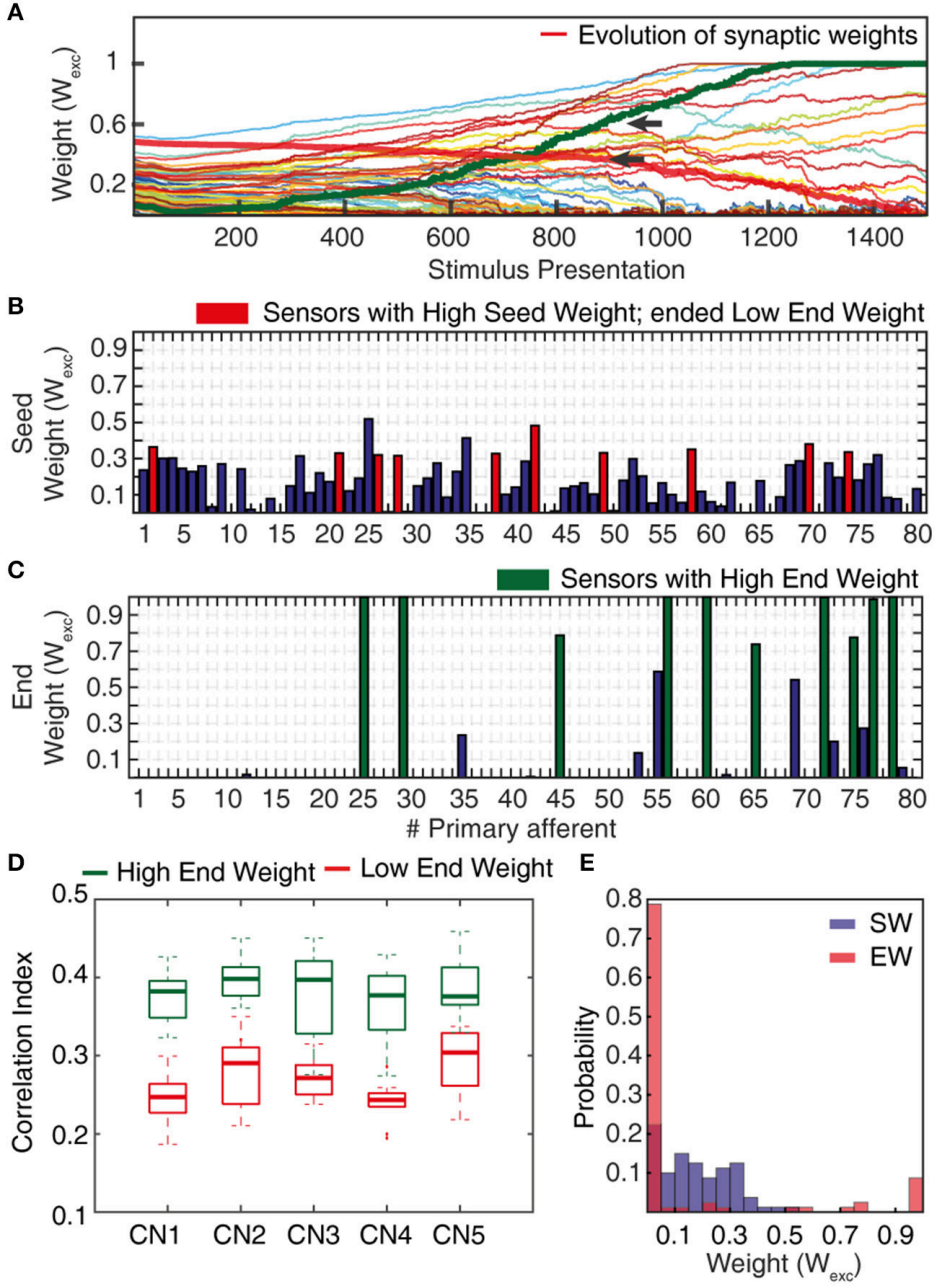
Synaptic weight changes during the learning process. **(A)** Evolution of the synaptic weights during a learning process of 1,500 stimulus presentations, with the evolutions of two highlighted synapses indicated by red and green thick lines. **(B)** Starting weights of the PA synapses (“seed weights”) for the sample learning process. Red bars indicate a set of 10 sensors/PA synapses that started with high weight but ended up as low weight synapses (“losers”). **(C)** The end weights of the PA synapses after a completed learning process. Green bars indicate the ten synapses with the highest end weights (“winners”). **(D)** Box plots of the correlation indexes for the end weight “winners” and for the end weight “losers” for each of the five seed weight configurations tested. The differences in correlation index between the winners and losers was statistically different in each of these five cases (*p* < 0.001, paired *t*-test). **(E)** Histogram of the distribution of synaptic weights before and after learning in the example CN (CN1).

In order to induce learning in the CNs, we used a series of 1,500 stimulus presentations. Each of the 1,500 stimuli hence corresponded to a spatiotemporal pattern of PA spike inputs (Figure 4), where all of the 80 PAs were activated during each stimulus presentation. The 1500 presentations were generated from the 5 physical stimulus presentations. Each stimulus presentation was hence repeated 300 times. Rather than feeding the system with identical spatiotemporal spike patterns for each of these 300 repetitions, we added a spike time jitter to all tactile sensor spike output responses. Gaussian noise with zero mean and standard deviation σ (σ = 5 ms) was added to the individual spike times of each PA. The motivation for this injection of noise rather than repeating identical patterns of PA spike responses was because we observed it to make the learning more robust. It also allowed an identification of the spread of learning outcome (end weight “landscapes”) for each model setting (Figures 5B,C).

**Figure 6:**
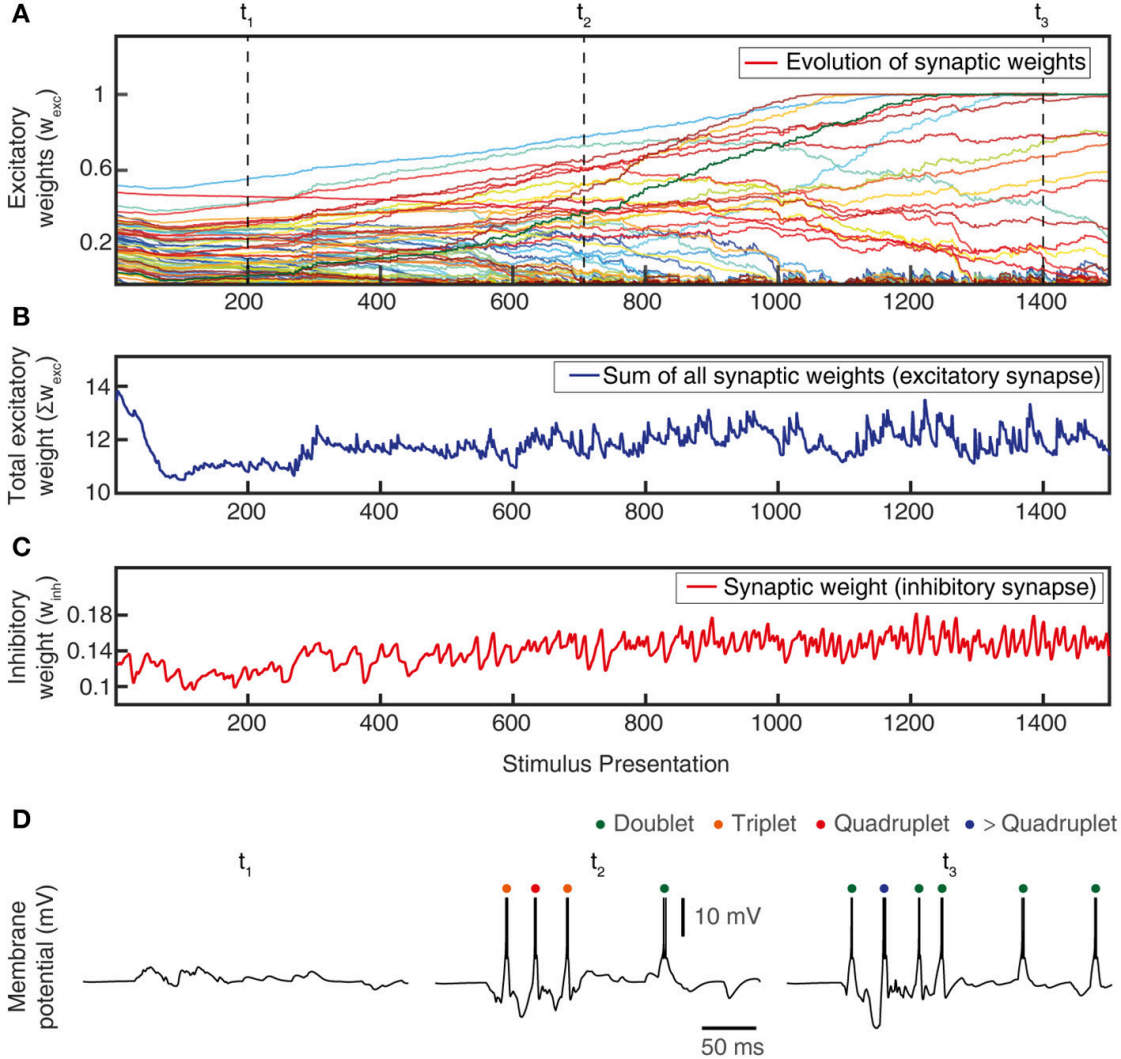
Total synaptic weights and response evolution during learning. Relationship between synaptic weight evolution during the learning process **(A)** and the sum of the excitatory synaptic weights **(B)** and of the inhibitory synaptic weights (equal for all inhibitory synapses) **(C)**. Note that the latter two parameters were controlled by the feedback mechanisms to ensure stability of the sum of the excitatory synaptic weights on the CN (by homeostatic plasticity) and of the total calcium activity (by the weight of the inhibitory synapses) (Figure 3B). **(D)** Example responses of the CN to the same stimulus condition (indentation 20 mm, see Figure 4) at three different time points during the learning process (indicated in **A**).

### Excitatory synaptic weight learning

During the learning process, the individual excitatory synaptic weights were gradually learnt, i.e., there was an alteration in the weight of each synapse. All the synaptic weights were updated after each stimulus presentation. The weight change for the individual excitatory synapse (*i*) is given by Equation 16.

(16) $$\begin{array}{r} wc_{exc,i}=\int_{t_{0}}^{t_{\max}}\left\{\left(A_{tot}^{Ca^{2+}}\left(t\right)-\left(Avg_{A_{tot}^{Ca^{2+}}}.Syn_{EQ}\right)\right).\;A_{loc}^{Ca^{2+}}\left(t\right)\right\}.\;K\;.\;dt \end{array}$$

The driving force for the net synaptic weight change (*wc*_*exc, i*_) is given by the integral of the correlation between main compartment total calcium ($A_{tot}^{Ca^{2+}}$) and local calcium activity for each synapse ($A_{loc}^{Ca^{2+}}$) from *t*_0_ to *t*_max_ (corresponding to the start and end of each stimulus presentation). The $A_{tot}^{Ca^{2+}}$ is offset to zero by the *Learning threshold* (Figure 3D), where $A_{tot}^{Ca^{2+}}$ above the Learning threshold is in the positive zone (potentiation) and below is the negative zone (depression) (similar to Graupner and Brunel, 2012). In other words, the main compartment calcium activity is a gate for the plastic reaction in the local synaptic compartment, deciding whether potentiation or depression should occur. Therefore, the product of the counterbalanced $A_{tot}^{Ca^{2+}}$ and individual synapse $A_{loc}^{Ca^{2+}}$ (Figures 3E–G) defines the net learning drive for each respective synapse as a function over time (Figures 3F–H). The value of the time integral of the net learning drive attained at *t*_max_ decides to what extent each specific synapse should be potentiated (Figure 3F) or depressed (Figure 3H). The strength of the potentiation/depression for that individual synapse is further multiplied with a constant given by a sigmoid function (Figure 3I) of the current synaptic weight (*w*_*exc, i*_), a constant which we called the synaptic weight compensation. This is motivated by that the insertion of a synaptic receptor ion channel in the synaptic membrane can be regarded as a chemical reaction, where the addition of a new channel will become less likely the higher the number of ion channels already inserted and vice versa. The sigmoid function is defined by $\;S\left(t\right)=\;\frac{1}{1+e^{-t}}\;$, with an arbitrarily chosen steepness gain of 0.005.

The *Learning threshold* is given by the product of the average total calcium ($Avg_{A_{tot}^{Ca^{2+}}}$) and synaptic equilibrium (*Syn*_*EQ*_) where $Avg_{A_{tot}^{Ca^{2+}}}$ is the mean of $A_{tot}^{Ca^{2+}}$ across the last three consecutive stimulus presentations. The averaging was required to avoid instability in the learning and could in principle correspond to the dampening effect of the time required in biological systems for protein synthesis and/or ion channel insertion. The *Syn*_*EQ*_ is a gain factor, used to attain a homeostatic synaptic plasticity (Turrigiano and Nelson, 2004; Turrigiano, 2011) to keep the sum of the excitatory synaptic weights in control. It is defined as a linear function of the total excitatory synaptic weight (∑*w*_*exc*_), with a dual slope having zero point preset to *SetPoint*_∑_*w*__*exc*__ (in our model, that point is set to 10). The slopes intercepts for this linear function are defined such that, when total excitatory synaptic weight is 10 the *Syn*_*EQ*_ is 1. When ∑*w*_*exc*_ is >10, the slope is 0.12, whereas for ∑*w*_*exc*_ < 10, the slope is 0.04. The differences in slopes were necessary to prevent neurons from being stuck in a depressed state, and to prevent unstable, rapid potentiation once above the set point. In principle, the slope differences can be regarded as corresponding to that the insertion and the removal of synaptic conductances in biology is handled by partly separate chemical reactions (Jörntell and Hansel, 2006).

### Inhibitory synaptic weight learning

In model CNs without adaptation of synaptic inhibition, we observed that synaptic weight changes in the PA synapses tended to make the CNs unstable, which degraded learning stability (Figure 10). Instability was attained gradually during the learning process and typically resulted in that either the CN had excessive calcium activity or no calcium activity at all. In such cases, the CN would often swing between these two extremities several times during the learning process. We found that it was important that the model system prevented the CNs from entering such unstable states and a main effective regulator was found to be gradual adaptation of the total inhibitory synaptic weight around a set point of the total calcium activity of the neuron.

Therefore, during the learning process, after each stimulus presentation, also the total inhibitory weight was adapted toward a set point, which was defined by the activity of the calcium spike rate, closely related to $A_{tot}^{Ca^{2+}}$. A high calcium activity during the stimulus presentations was countered by an increase in total inhibitory synaptic weight, whereas a low calcium activity was countered by a decrease in this weight. A plastic regulation of the weight of inhibitory synapses against postsynaptic calcium activity or postsynaptic calcium-dependent bursting activity has previously been found in various neurons across the brain (Kawaguchi et al., 2011; Hulme and Connelly, 2014; Lourenço et al., 2014). In our model, we found this feature to prevent overexcitation in the simulated CNs as the learning of the synaptic weights progressed. A function with dual linear slope was used to define the rate of *inhibitory weight change* with respect to the calcium spike rate. Inhibitory weight change is summed with the existing inhibitory synaptic weights (*w*_*inh*_). The *calcium activity set point* ($SetPoint_{Ca^{2+}}$) (Table 1) is the critical factor that defines the calcium spike rate, around which the inhibitory weight adapts to keep the system in balance. These slope functions are built zeroing from the preset point ($SetPoint_{Ca^{2+}}$), where the range of weight change is set between −0.01 and 0.01 for calcium spike rates between 0 and 200 Hz, respectively. In order to dampen the rate of adaptation, we used a moving average of the calcium spike rate across the last three stimulus presentations as the input to the adaptation.

Adaptation of the total inhibitory synaptic weights was implemented because the change in the weights of the excitatory PA synapses on the CN neuron could lead to large changes in the levels of the LVA (voltage-activated calcium channel) activity, which in turn led to unstable learning (as in Figure 10). Adaptation of the inhibitory synaptic weights balanced the LVA activity and resulted in a maintained stable learning process. All adaptation of the weight of the synaptic inhibition was evenly distributed across all the inhibitory synapses. Calcium-dependent potentiation of inhibitory synapses has previously been found to be confined to the inhibitory synapses that were active during the calcium activity (Kawaguchi et al., 2011; Hulme and Connelly, 2014; Lourenço et al., 2014)–indeed, in our simulated set of inhibitory synapses, all of them were active under all stimulus presentations as described above.

### Variations of initial intrinsic CN parameters

In addition to testing the effects of excitatory synaptic seed weight distributions on the outcome of the learning process (see below) we also wanted to test the effects of intrinsic CN parameters. Moreover, the values of these parameters to some extent relied on assumptions, and we wanted to know the sensitivity of the model to these assumptions. Therefore, in separate simulation runs, some of the intrinsic CN parameters were varied as described by Table 1. All of the initial parameters were kept constant during the learning process.

## Model data analysis

### Correlation index

The *correlation index* measure (Figure 5D) was used to quantify the degree of correlation between the spiking activities of two or more PAs while being provided with sensory inputs. We compared PAs whose synapses after learning ended up as High End Weight (HEW) synapses with high seed weight synapses that ended up as Low End Weight (LEW) synapses.

To compute this measure, we considered 10 HEW (green bars in Figure 5C) and 10 LEW synapses (red bars in Figure 5B). We evaluated the spike trains for these 20 PAs across all the five stimuli (Figure 4) by means of Victor-Purpura distance (VPd) (Victor and Purpura, 1996) spike train metrics. VPd gives a measure of the similarity between two spike trains by measuring the minimum cost necessary to transform one spike pattern into the other. The distance calculation is based on two rules, (i) adding/deleting a spike (*cost* = 1) and; (ii) shifting the spike by an interval of Δt (*cost* = *q*. Δ*t*, in our simulation *q* = *10/s*). By making comparisons between all the primary afferent spike trains evoked by different stimuli, we obtained a matrix of comparisons between all of the stimuli used (Rongala et al., 2017). For each neuron configuration, these matrices were used to compute the correlation index value for sensors with HEW synapses (Figure 5C, green) as given by computing the average VPd across all the 10 synapses with HEW for all the 5 stimuli. The same procedure was carried out to calculate the correlation index value for sensors with LEW synapses (Figure 5C, red). The difference between correlation index values of LEW and HEW sensors was the *correlation index difference* (Figure 13).

### Correlations between synaptic responses

**Figure 7:**
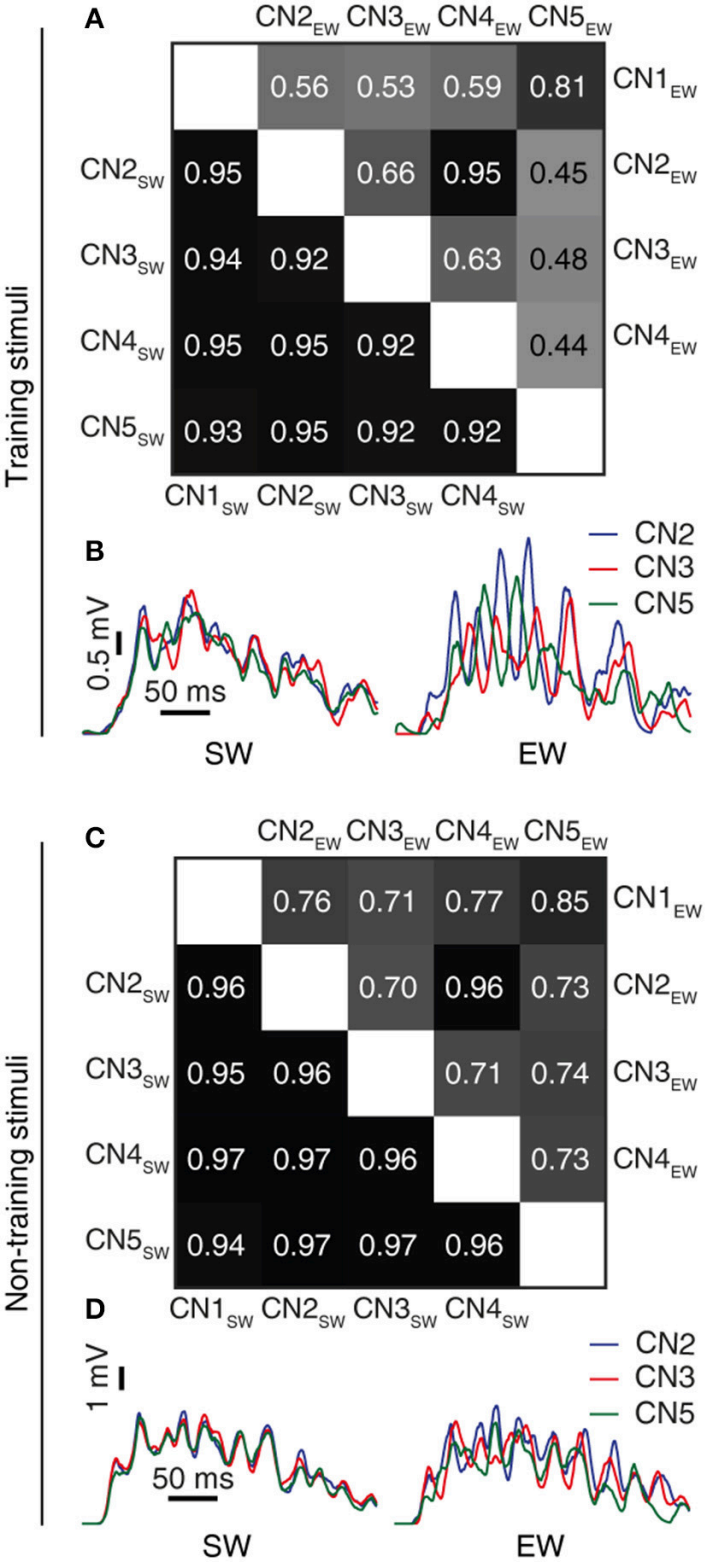
Learning resulted in decorrelated responses between neurons to the same stimulus. **(A)** Triangle matrix for the correlations between synaptic responses evoked by the same training stimulus before (CN1_SW_-CN5_SW_) and after learning for all five cuneate neurons (CN1_EW_-CN5_EW_). The difference in correlation between the two groups was statistically significant (*p* = 0.001, Wilcoxon signed rank test) **(B)** Averaged synaptic responses to the stimulus (‘indentation 20 mm') repeated 100 times for three different CNs (CN2, CN3, CN5) before and after training. **(C,D)** Similar displays as in **(A,B)** but for a novel stimulus that was not part of the training set (“indentation 10 mm;” Oddo et al., 2017). The difference in correlation before and after learning was statistically significant (*p* < 0.001, Wilcoxon signed rank test).

**Figure 8:**
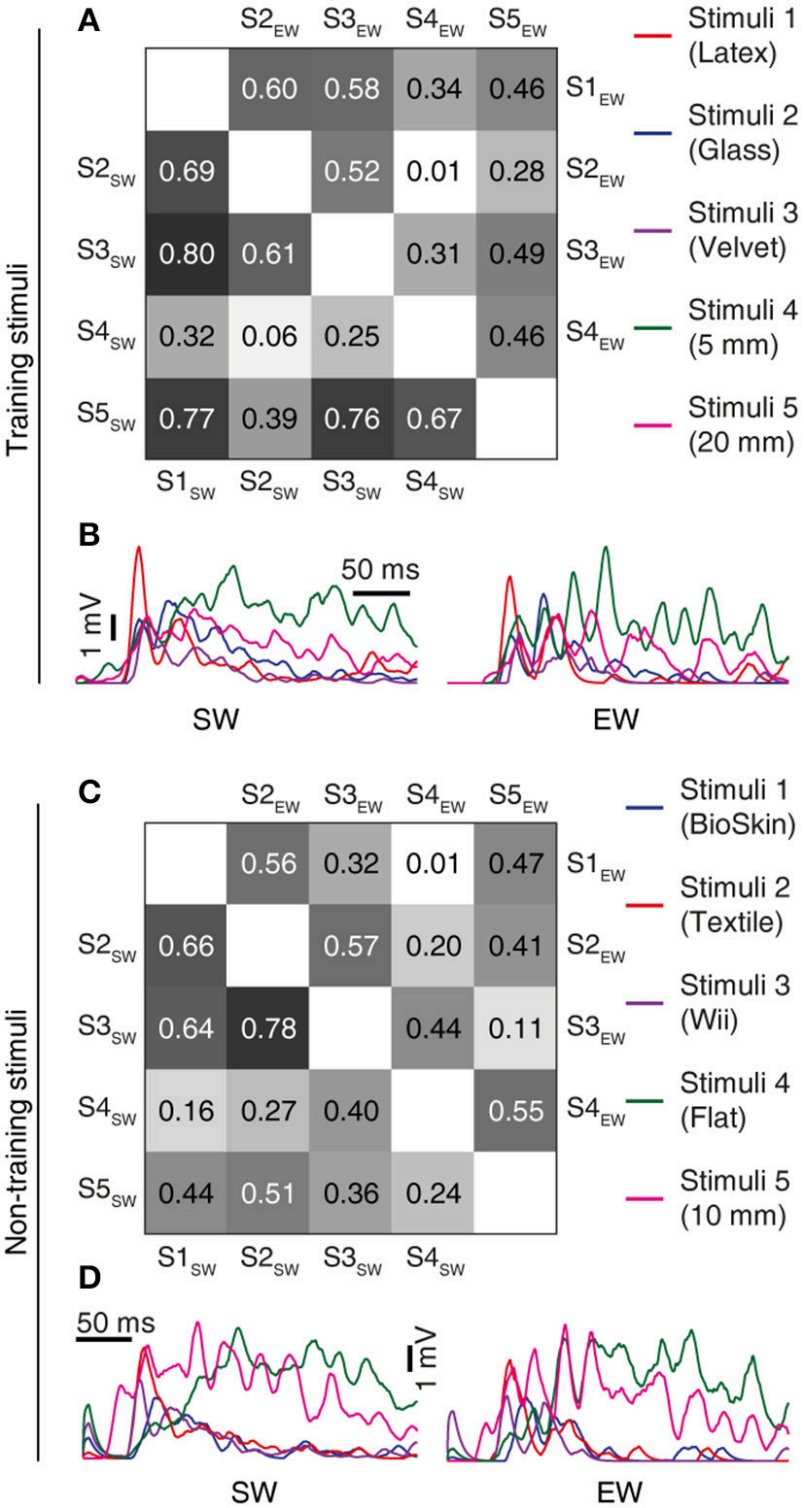
Learning resulted in decorrelation of the responses to different stimuli in the individual CN. **(A)** Triangle matrix for the correlations between synaptic responses evoked by the training stimuli before and after learning in the same CN. Otherwise the display is similar to Figure 7A. The difference in correlation before and after learning was statistically significant (*p* < 0.001, Wilcoxon signed rank test) **(B)** Averaged synaptic responses of the CN (CN1) to the full set of training stimuli. **(C,D)** Similar display as in **(A,B)** but for non-training stimuli previously reported for shapes (Oddo et al., 2017) and for textures (Rongala et al., 2017). The difference in correlation between the two groups was statistically significant (*p* = 0.028, Wilcoxon signed rank test).

The correlation measure (Figures 7, 8) was used to compute how correlated the synaptic responses were across different learning conditions. To compute this measure, we consider all the 80 synapses. We convolute the spike trains of all 80 PAs using the same EPSP time constants as in CN *in vivo* recordings (Bengtsson et al., 2013). Further we multiply each of these convoluted signals with the respective synaptic weights of the corresponding PA. The sum of all the 80 weighted convoluted signals was the simulated intracellular signal displayed (Figures 7, 8). The correlation between two simulated intracellular signals is computed with the inbuilt MATLAB® function *xcorr* (with zero lag).

### Multi-dimensional scaling

**Figure 9:**
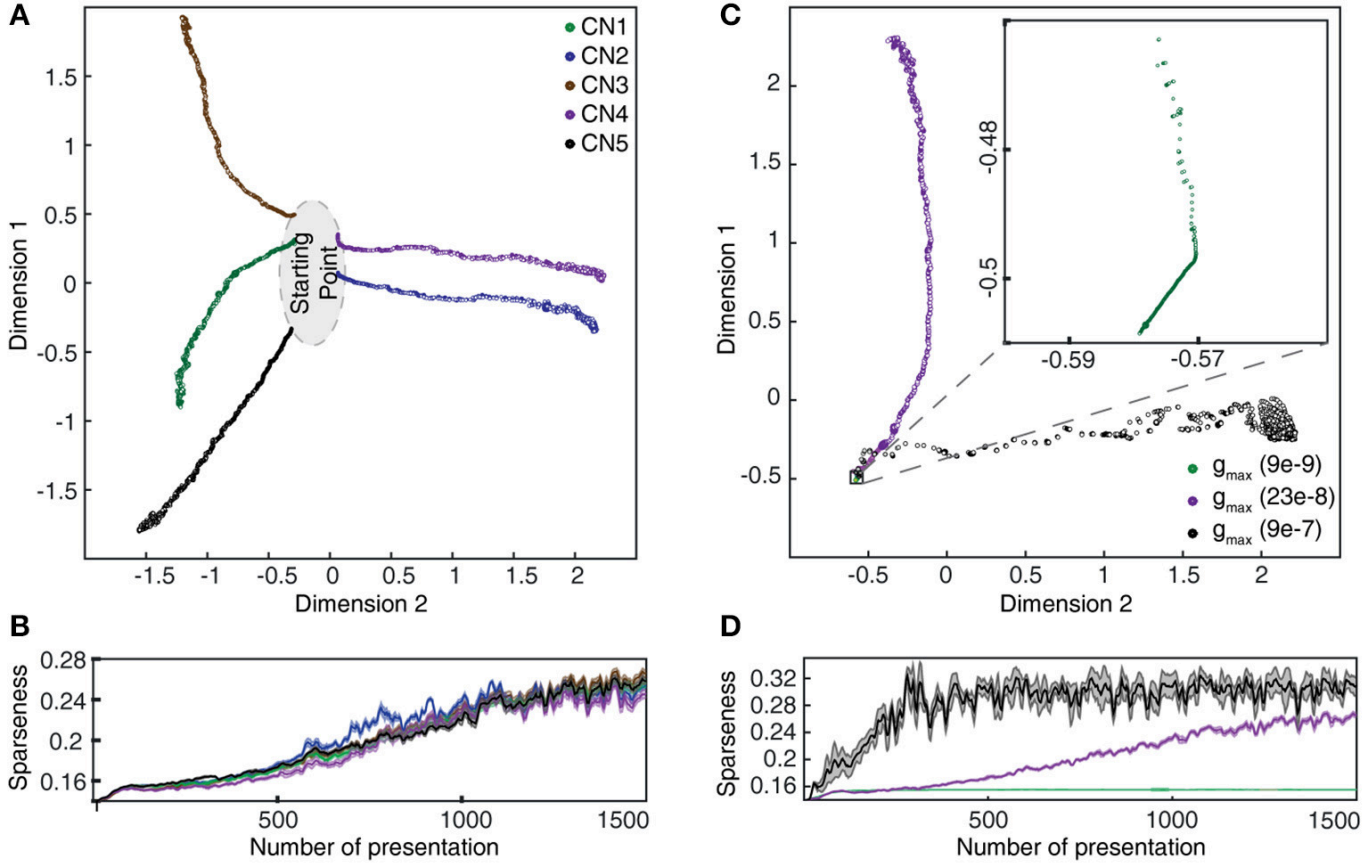
Evolution of synaptic weight landscapes and sparseness during the learning process. **(A)** Evolution of the synaptic weight landscapes during the learning process visualized by MDS for the five seed weights tested. **(B)** Corresponding evolution of the sparseness of the synaptic weight distributions. **(C)** Dependence of the evolution on gmax for seed weight #1. The intermediate value was the default configuration for this parameter. **(D)** Corresponding plot for the sparseness.

**Figure 10:**
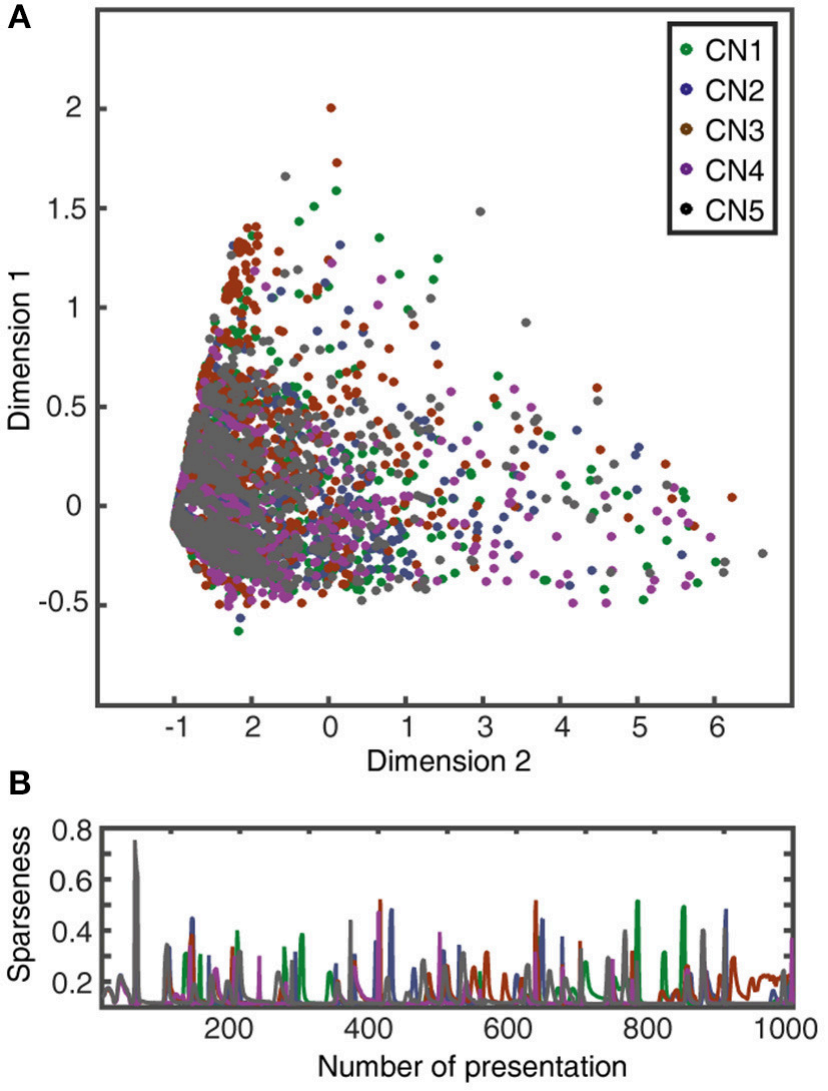
Learning without supralinear calcium responses. **(A)** Evolution of the synaptic weight landscape for a CN without supralinear calcium activity components. **(B)** Corresponding evolution of the sparseness of the synaptic weight distribution (cf. Figure 9B).

To further illustrate the specific distribution of synaptic weights, we used the Classical multi-dimensional scaling (MDS) method (cmdscale, an inbuilt MATLAB® function). The distribution of weights (*w*_*exc*_) across the 80 PA synapses is here denoted the synaptic weight landscape. Using MDS, the differences between the synaptic landscapes of two or more CNs were visualized as distances in two-dimensional displays (Figures 9A,C,10A). The input distance vector to multi-dimensional scaling is calculated as a simple Euclidian distance between synaptic weight landscapes.

### Computation of the sparseness of the synaptic weight distribution

We also measured the degree of dispersion of synaptic weights, or the sparseness of the synaptic weights, among the 80 available PA synapses (Figures 9B,D, 10B). For this purpose, we took the measured *synaptic weight sparseness* using the ratio between the *l*_2_ and *l*_1_ norm of the weight vector (Yin et al., 2014). This measure will report its minimal value when all synapses have exactly the same weight and a value of one when one synapse has maximum weight and all other synapses have zero weight. It was used to track the evolution of the synaptic weight dispersion during consecutive stimulus presentations and was therefore also an indicator of the stability of the learning.

### Statistical tests

After the learning process, the correlation index of the High End Weight (HEW) PA synapses (*N* = 10) was compared to that of the Low End Weight (LEW) PA synapses (*N* = 10) for each CN configuration using paired *t*-test (these distributions were on visual inspection comparable to normal distributions, see Figure 5D). The changes in correlation between the synaptic responses, of different CNs to the same stimulus and of the same CN to different stimuli, induced by training were statistically quantified using the paired, two-sided Wilcoxon signed rank test.

## Results

### Inhibitory synaptic inputs and intrinsic responses of cuneate neurons *in vivo*

To support the construction of our model, we started out by extending a previous characterization of the projection neurons of the cuneate nucleus in the decerebrate cat (Bengtsson et al., 2013) to also include their inhibitory synaptic inputs and intrinsic membrane responses, using a set of *in vivo* whole cell intracellular recordings from projection neurons (*N* = 15) and interneurons (*N* = 8) (Figure 1A). A touch to the receptive field of a cuneate projection neuron activated both excitatory and inhibitory synaptic inputs (Figures 1A,B) and the interneurons, which are responsible for synaptic inhibition evoked by skin stimulation in this preparation (Bengtsson et al., 2013). Spontaneous unitary inhibitory postsynaptic potentials (IPSPs), presumably driven by spontaneous interneuron spiking, were very small (Figures 1B,C) (the average median IPSP amplitude was −0.55 ± 0.08 mV, recorded from *N* = 15 cuneate projection neurons). In fact, this amplitude is an overestimate since many of the apparent IPSPs were too small to be detected from baseline noise using our template based identification method. In contrast, maximal evoked compound IPSPs (evoked from outside the receptive field of the excitatory input, see Bengtsson et al., 2013) were large (−14.3 ± 2.1 mV) and recruited in a continuous, gradable fashion (Figure 1D). Such maximal compound IPSPs had a response onset latency of 6.1 ± 0.7 ms, which is about 1.0 ms longer than the latency time of primary afferent EPSPs in the same type of preparation (Bengtsson et al., 2013). This observation strongly suggests that the compound IPSPs were due to primary afferent activation of the inhibitory interneurons. Together, these findings indicated that a high number of interneurons (>30, but possibly 100's) innervated each projection neuron with relatively uniformly low weighted inhibitory synapses.

A strong involvement of intrinsic voltage-activated conductances in shaping the membrane potential trajectory over time was suggested by the subthreshold spontaneous activity of the cuneate projection neurons recorded near resting potential (Figure 1E). Note the doublets, triplets or quadruplets of spike firing, which were observed in all projection neurons, but none of the interneurons. Occasional single spike firing events dramatically altered the spike afterhyperpolarization phase (red trace in Figure 1E), which further suggested the presence of intrinsic membrane potential dynamics. Indeed, using current clamp to generate temporary hyperpolarization of the membrane potential resulted in strong rebound excitation (Figure 1E), which demonstrates the contribution of such active intrinsic conductances. Similar intrinsic responses were recorded in all cuneate projection neurons when hyperpolarized by at least −200 pA for at least 200 ms (*N* = 15). Since such intrinsic responses can be expected to be an important part of defining the time course of calcium influx into the neuron, which in turn will be important for its learning process during a time-varying synaptic input, an important component of our full model below was the “intermediate dynamics” model of the of the cuneate projection neurons. We observed that there were relatively wide differences in the intensity and the speed of the intrinsic responses (Figure 1E) between the cuneate projection neurons. Therefore, we designed the intermediate dynamics model of these membrane potential dynamics against one of our recorded cuneate projection neurons, with the primary aim of approximating its overall dynamics across a range of conditions rather than focusing on capturing its behavior under any single condition exactly (for the full model, we later also will show model experiments in which parameters influencing the intermediate dynamics are adjusted to modify the intrinsic responses of the cuneate projection neurons, showing that model behavior was robust across a range of intermediate dynamics). Our intermediate dynamics model approximated the observed membrane potential dynamics well across a range of hyperpolarizations (Figure 1F). The model also resulted in a reproduction at least of the overall dynamics for responses evoked by touch (Figure S1).

In order to verify that the recorded IPSPs (Figure 1) could be generated by the local interneurons, we made intracellular recordings also from these neurons. Intracellular recordings were identified as projection neurons and interneurons based on their characteristic spike shapes (Figure 2A). The spontaneous spike firing frequencies were 11 ± 4.8 Hz (mean ± sd.) vs. 8.8 ± 2.2 Hz for projection neurons (*N* = 15) and interneurons (*N* = 8), respectively. Interneurons responded well to touch to the skin (Figure 2B) and on electrical skin stimulation (Figure 2C). The EPSPs evoked by electrical skin stimulation in the interneurons were considered monosynaptic based on the response latency time and the low variability of the EPSPs (Bengtsson et al., 2013; Figure 2C). Based on a qualitative analysis of the extent of their receptive fields on the skin (Figure 2B), interneurons were found to have much wider excitatory receptive fields than the cuneate projection neurons (which were previously quantitatively defined in Bengtsson et al., 2013). Combined with the rapid responses of the interneurons to PA synaptic input (Figure 2C), these observations made it plausible that their activation was responsible for the compound IPSPs recorded in the cuneate projection neurons on stimulation within or in the vicinity of their receptive fields (Figure 1D). The apparent absence of intrinsic regenerative responses to skin stimulation in the interneurons (Figure 2B) suggested the function of the cuneate interneurons to be a linear transfer of PA input, inverted to IPSPs, to the projection neurons. These findings motivated our simplification of the inhibitory synaptic inputs to the cuneate projection neurons to be modeled as a set of unitary weight inhibitory synaptic inputs directly generated by the spikes of the individual PAs (Figures 3A,B).

### Functional structure of the CN model

In our integrated cuneate model, the biological sensorized skin was replaced with a bionic fingertip (Oddo et al., 2016; Figure 3A and Figure S2). The model cuneate projection neuron (CN) was composed of the intermediate dynamics model described above (Figure 1F), that implemented input- and time-varying intrinsic responsiveness, and the excitatory and inhibitory synaptic inputs from the sensors that drove the activation of the intermediate dynamics of the CN (“Total calcium activity” in Figure 3B). The factor *g*_max_ allowed an adjustment of the ratio between the synaptic conductances and the background, leak, conductance, which is later in the paper used for simulating CNs with different input resistances or sizes. The rest of the diagram in Figure 3B indicates adaptation mechanisms for the excitatory and inhibitory synaptic weights, which implemented a Hebbian-type of learning combined with synaptic weight scaling as described below.

Depending on the correlation between the calcium activity in the main compartment and in the individual synaptic spaces (Figures 3C,D), the weight of each excitatory synapse was updated after each stimulus presentation (Figures 3E–H). To illustrate this process, Figure 3E shows that the local synaptic calcium activity crossed the learning threshold twice in this example. But for calculating the learning signal, also the calcium activity in the main compartment (Figure 3D) was taken into account. For the first episode of suprathreshold calcium activity in the synapse (Figure 3E), the calcium activity in the main compartment was in the “positive zone,” i.e., sufficiently high to permit potentiation of the synaptic weight. Therefore, this first episode made the net learning drive of this synapse positive (Figure 3F). The second episode of suprathreshold calcium elevation in the synapse (Figure 3E) instead coincided with calcium activity in the main compartment being partly in the “negative zone” (Figure 3D). Therefore, the net learning drive for this episode was partly negative (Figure 3F). The net learning effect of each stimulus presentation and each synapse was the integral of all of these episodes, which in this case ended up positive (Figure 3F). For another synapse, the suprathreshold calcium activity (Figure 3G) mostly coincided with main compartment calcium activity being in the negative zone (Figure 3D), which resulted in a negative integral of the net learning drive (Figure 3H).

For each update of synaptic weights, the weight of the synapse itself was an additional factor used to scale the synaptic weight change (Figure 3I; synaptic weight compensation in Figure 3B) (Bi and Poo, 1998). Altogether, this architecture resulted in a Hebbian form of plasticity (Hebb, 1949). The end result was a form of plasticity that had properties being related to spike-time dependent plasticity (STDP) (Markram et al., 1997; Graupner and Brunel, 2012) but being less sharply dependent on the exact spike timing (Helias et al., 2008). The motivation for taking this approach to formulate the driving forces for the learning rule was to let the dynamics of the cellular learning signal, and its shaping by the integration of peripheral synaptic input have its origin in biological data recorded from cuneate projection neurons *in vivo* (Figures 1E,F) rather than in assumptions made for other learning systems. We believe that overall, net learning effects are comparable to those obtained under some settings of those previous models (Graupner and Brunel, 2012) (Discussion) but more specifically constrained to the effects that might arise in the cuneate projection neurons.

The integrated model also used two separate feedback regulatory systems. First, the total inhibitory synaptic weight was updated based on the total calcium activity, according to a fixed set point (Figure 3B). Adaptation of the total inhibitory synaptic weights was implemented because we found that the change in the weights of the excitatory PA synapses on the CN neuron could lead to large changes in the levels of activity in the voltage-activated calcium channels, which in turn led to unstable learning. A plastic regulation of the weight of inhibitory synapses against postsynaptic calcium activity or postsynaptic calcium-dependent bursting activity has previously been found in various neurons across the brain (Kawaguchi et al., 2011; Hulme and Connelly, 2014; Lourenço et al., 2014).

Secondly, *SetPoint*_∑_*w*__*exc*__ is a control signal used to implement a homeostatic synaptic plasticity mechanisms (Turrigiano and Nelson, 2004; Turrigiano, 2011) through which the sum of the excitatory synaptic weights is kept under control. The total excitatory synaptic weight was driven toward this set point by adjusting the learning threshold for synaptic potentiation vs. depression (Figure 3B; positive vs. negative zone in Figure 3D). Hence, the propensity for LTP or LTD was affected by the current total synaptic weight and the effect of the learning threshold was therefore a synaptic weight scaling. The set point of the learning threshold (Figure 3B) is the fixed sum of the excitatory synaptic weights toward which the adaptation of the learning threshold strives to bring the system. Further details and motivations are given in Methods.

### Synaptic weight changes during the learning process

The sensorized bionic fingertip was used to provide the CN with training data. The bionic fingertip provided the important feature of a system of PA sensors where there is a consistent relationship between the activation of the different sensors across different conditions or tactile experiences. The CN learning process consisted of 1,500 presentations of five training stimuli, each activating all of the 80 PA sensory inputs but in different spatiotemporal patterns (Figure 4), presented in a pseudorandom order. The training stimuli consisted in three touch-an-slide conditions and in two dynamic indentation stimuli (Figure 4). During the learning process, the gradual transition from initial, or “seed,” synaptic weights to the final distribution of synaptic weights (“end weight”) was monitored (Figure 5A). Interestingly, the weight of a synapse could for example evolve from a very low seed weight to a high end weight (green thick line), or from high seed weight to near-zero end weight (red thick line) and any intermediate or mixed development. Such examples showed that the fate of an individual synaptic weight during the learning process was not depending on its starting point but suggested some underlying functional principle.

The learning process resulted in a transformation of the initial random distribution of low-moderate weight synapses (seed weight, SW) (Figure 5B) to a highly skewed distribution with a few high weight synapses (end weight, EW) (Figure 5C), similar to cuneate neurons in adult animals (Bengtsson et al., 2013). We simulated CNs with five different pseudo-randomized SWs, which below are referred to as CN1-5, based on the assumption that initial random synaptic weight distributions likely differ between biological cuneate neurons in the same animal. For each CN, there was at the end of the learning process a unique end weight distribution (EW), dominated by different specific synapses with high weights (Figure S3). The seed weight losers (red bars in Figure 5B) differed from the end weight winners (green bars in Figure 5C and Figure S3) in that they conveyed sensor spiking activity that was less correlated across the training stimuli (Figure 5D), as measured by the Victor-Purpura distance metric for comparisons between spike trains. During the learning process, the overall adaptive regulation of the total excitatory and inhibitory synaptic weights in the model (Figure 3B) helped stabilizing the activity of the CN (Figure 6), which was important for a stable learning outcome. In addition, in parallel with the acquisition of highly skewed synaptic weight distributions, the cuneate neurons also gradually acquired a firing that was dominated by brief, episodic bursts of spike output, similar to the firing mode observed for these neurons in adulthood *in vivo* (Bengtsson et al., 2013; Figures 1, 6D, Figure S1).

### Learning resulted in decorrelations of synaptic inputs between neurons and stimuli

The next question asked was what functional effects the learning process resulted in. We found that between the individual CNs, the learning process resulted in a decorrelation of the initially highly correlated temporal patterns of the synaptic responses to each given training stimulus (Figures 7A,B). Hence, a consequence of the learning process was an increased differentiation of the CN responses to a given input, which was statistically significant at *p* ≤ 0.001 (Wilcoxon signed rank test). In addition to testing the stimuli that the CNs were trained to, we also used a set of previously non-encountered stimuli, consisting of three types of texture sliding and two touch indentations, here referred to as non-training stimuli. Similar to the case with the training stimuli, the learning process resulted in a decorrelation of the synaptic responses of the CNs also to the non-training stimuli (Figures 7C,D; *p* < 0.001, Wilcoxon signed rank test). Moreover, in the individual CN, whereas the differences in the spatiotemporal patterns of PA input between stimuli to some extent generated specific synaptic responses already in the “naïve” state, the responses became markedly differentiated with learning (Figures 8A,B). For each of the five CNs, CN1-5, the learning process resulted in a statistically significant decrease in the correlation between the synaptic responses to the five training stimuli (*p* < 0.001, Wilcoxon signed rank test). Such synaptic response differentiation is a basis for tactile input feature segregation (Jörntell et al., 2014) and the mechanism described here can hence be expected to improve the resolution at which the neuronal segregation of spatiotemporal PA sensory input patterns occurs. The learning process also resulted in that the individual CN increased the segregation of its responses to different non-training stimuli (Figures 8C,D). For the illustrated CN, the learning process resulted in a statistically significant decrease in the correlation between the synaptic responses to the five training stimuli at *p* = 0.027 whereas the p values for the other four CNs were below 0.001 (Wilcoxon signed rank test). The results with non-training stimuli hence indicated that the acquired learning was generalizable to previously non-encountered experiences, which is difficult to achieve in traditional pattern recognition systems (Spanne and Jorntell, 2015).

### Evolution of synaptic weight landscapes and sparseness during the learning process

The gradual evolution of the synaptic weight distributions (or their “landscapes”) during the learning process was further studied by plotting the weights of all the 80 PA synapses of each CN in an 80-dimensional space, embedded in 2D plots using multi-dimensional scaling (MDS). First, the analysis showed that the end weight landscape, which is the basis for the segregation of responses between neurons and inputs shown in Figures 7, 8, depended on the seed weight landscape of the CN (Figure 9A). Secondly, it showed that the evolution of the synaptic weight landscape initially accelerated but later decelerated to hover around a relatively stable end point (Figure 9A), suggesting that the learning was self-stabilizing. Self-stabilization is further quantified in Figure 9B, which shows the temporal evolution of the sparseness of the synaptic weight distribution. This self-stabilization was lost on removal of the supralinearity in the calcium dynamic responses and in the local synaptic calcium activity (Figure 10). In this case, the main compartment calcium was the sum of the local calcium activity in all the synapses, i.e., without intrinsic calcium dynamics in the main compartment and without supralinear local calcium activity subsynaptically. Under such conditions, the learning process became chaotic, without any stable end point and without any development of sparseness of the synaptic weight distribution (cf. Figure 9A).

As we will describe in greater detail further below, we found that not only the seed weight landscape but also the intrinsic CN properties affected the outcome of the learning process. Of particular interest in relation to the effects of the calcium dynamic response (Figure 10) was the coupling between the synaptic input and the intermediate dynamics model of the CN, since the intermediate dynamics model governed the time course of the calcium signal and thereby the learning process. This coupling was strongly affected by the Maximum synaptic conductance constant (*g*_max_), which was the ratio between the maximum synaptic conductance and the leak conductance of the neuron. A decrease in this ratio, or coupling, substantially reduced the learning rate whereas an increase instead caused the learning to become rapid, unstable and with dramatic shifts, unlike the more gradual process when the learning system was in balance (Figure 9C). Interestingly, even in the setting with high *g*_max_ the system found a relatively stable end point in the synaptic weight landscape but with a different location compared to the basic configuration (Figures 9C,D). However, a high learning speed, or learning rate, may not be beneficial if the aim is to allow the result of the learning process to take a variety of experiences into account, which in turn could limit the range of conditions and novel experiences that the learnt network structure would be applicable to. To explore this, we tested the effects of “monotypic” sensory training.

### Importance of varied sensory experiences

**Figure 11:**
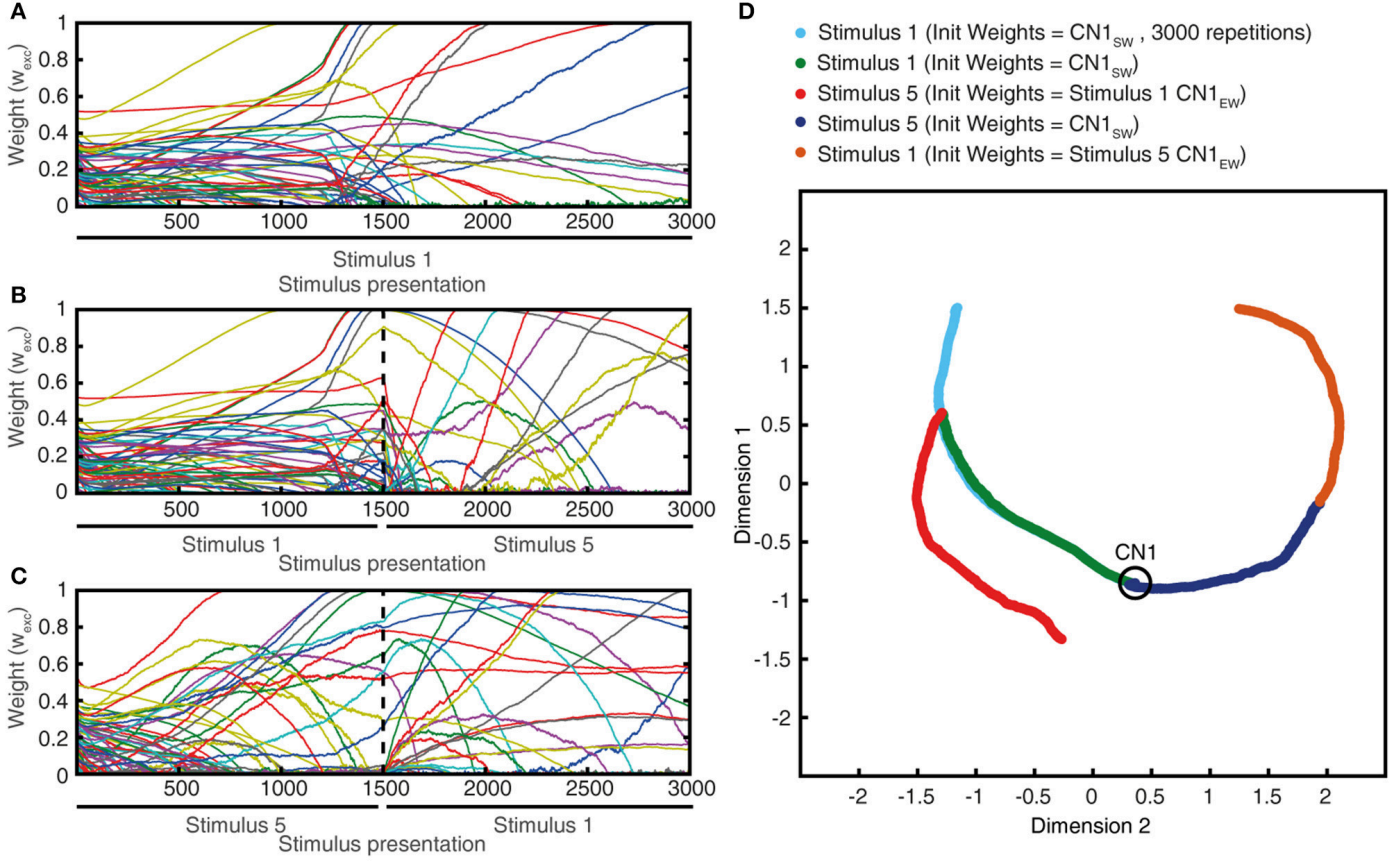
The effects of monotypic sensory experiences. **(A)** The evolution of the synaptic weight distributing using solely stimulus #1 (latex, Figure 4) as the training stimulus for 3,000 repetitions. **(B)** Similar experiment, but after 1,500 repetitions, stimulus #1 was replaced with stimulus #5 (indentation, 20 mm). **(C)** Similar experiment as in **(B)**, but now the 1,500 repetitions of stimulus #5 preceded the 1,500 repetitions of stimulus #1 (reverse order). **(D)** Synaptic weight landscape evolutions of the three experiments shown in an MDS plot.

Monotypic sensory training is here referring to a situation where the same sensory experience, or stimulus, is repeated a high number of times in succession. i.e., instead of using the regular learning process protocol with 1,500 presentations of the set of five stimulus presentations mixed in a pseudo-random order, here we ran the learning process with one single type of stimulus for the whole learning process. Similar to diversified sensory training (Figure 9A), monotypic sensory training also resulted in gradual changes in the synaptic weight landscape (Figure 11A). However, the type of stimulus applied had a decisive influence on the direction of development of the synaptic weight landscape (Figures 11A–C), and that a switch from one monotypic stimulus to another could sharply turn the evolution of the synaptic weight landscape in a new direction (Figures 11B–D). Also, with monotypic stimulus presentations, stable end points of synaptic weight landscapes were not clearly identified, in contrast to the relatively stable end points obtained when a mixture of stimuli was used in the learning process (Figure 9A). Indeed, instead of reaching a stable end point well before 1,500 trials (as in Figure 9A), monotypic sensory training instead could result in rapid changes in synaptic weight distribution at this time point (Figure 11A), which on inspection was found to be due to that the calcium activity of the CN could not be held within its set point and started to generate chaotic behavior (as in Figure 10). Hence, the order of presentation of the sensory experiences influenced the outcome of the learning process (Figure 11D), which indicates that the presence of a mixed, diversified set of sensory experiences over time will influence the result of the learning process. In addition, these findings also suggest that a high learning speed as in Figures 9C,D would make the CN more sensitive to individual sensory experiences and their order of presentation than in the basic configuration (Figures 9A,B).

### The intrinsic CN properties profoundly influenced learning outcome

**Figure 12:**
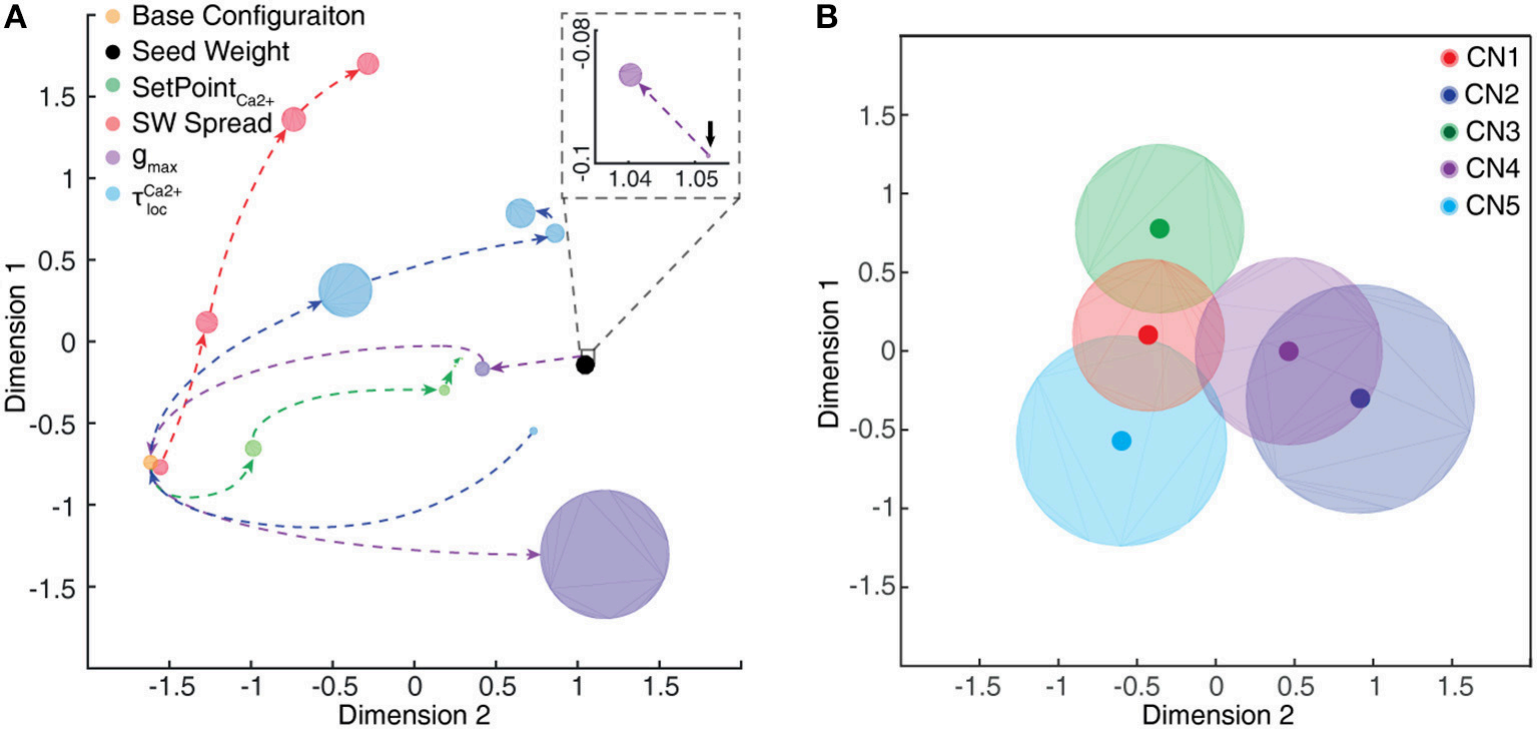
The intrinsic CN properties worked as additional seed factors. **(A)** For the seed weight #1, the end weight landscapes for all tested values of the intrinsic CN parameters, with the radii indicating the standard deviations across five repetitions of the learning. **(B)** The end weight landscape standard deviation for the whole range of intrinsic parameters is plotted for each seed weight. Note that the scale axes values are unrelated between the MDS plots.

Whereas the above analysis (Figures 5–8, Figure S3) indicated that the seed synaptic weight distribution was an important factor influencing learning outcome, i.e., which set of correlated sensors the CNs picked to selectively strengthen, also the intrinsic CN parameters could conceivably act as a seed factor. Morphological differences between cuneate neurons (Fyffe et al., 1986) is an example of a source of variability in intrinsic neuronal behavior, the level of expression of different ionic conductances another. To explore this issue, we tested to separately vary the time constant of the calcium signal in the local synaptic space, the coupling between the synaptic input and the intrinsic dynamics (already shown in Figures 9C,D) and the set point of allowed calcium activity in the intermediate dynamics model (which was controlled by adapting the gain of the inhibitory synaptic input, see Figure 3B). All intrinsic CN parameters, for all values tested (Table 1) influenced the outcome of the learning in terms of the end weight landscape (Figure 12A). Given that these intrinsic parameters influenced the behavior of the intermediate dynamics model and the calcium signal, either on a per stimulus presentation basis or over longer time, this was in itself perhaps not a surprising finding. However, the impact of these intrinsic parameters was profound and almost of comparative magnitude to the impact of the synaptic seed weight landscapes. The only apparent exceptions were the two lowest values of the *g*_*max*_ (inset) and the lowest τ for the calcium activity in the local synaptic space (triangle) (Figure 12A). However, the effect of these latter settings was that they reduced learning speed to a minimum and consequently resulted in little change from seed weight (black dot in Figure 12A). To estimate the relative stability of the learning, each configuration was run through the learning process five different times where each process was different as a minor noise was always injected in the sensor spike trains. The highest *g*_*max*_ value, with the fastest learning, resulted in the largest variability between learning processes (star in Figure 12A). All other parameter values were associated with lower variability in the learning outcome.

**Figure 13:**
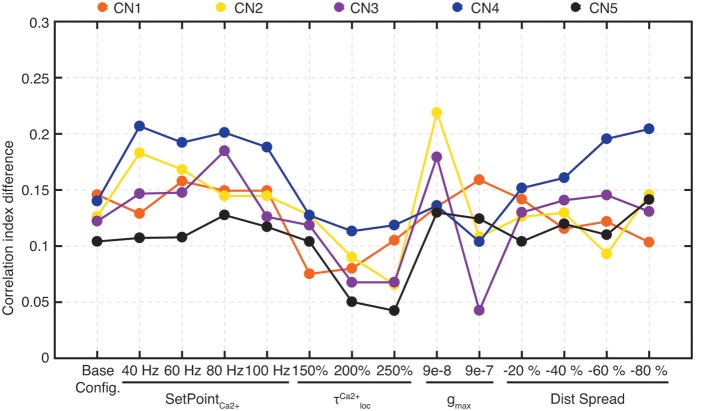
Improvements in correlation indexes for learning with different seed factors. The differences in correlation index between end weight winners and end weight losers (see Figure 5) plotted for all values of intrinsic CN parameters and all synaptic seed weights. Note that for some values of intrinsic parameters (gmax of 23e-9 and gmax of 9e-9, and τ for the local calcium activity of 50%) no learning took place (Figure 12) and the learning effects of those intrinsic parameter settings are hence omitted from this figure. Based on five CNs and 14 conditions, statistical comparisons were made for 70 conditions. The differences in correlation index between the end weight winners and losers was statistically different in each of these 70 cases (*p* < 0.05, paired *t*-test), except one (*p* = 0.11 for CN3 at gmax of 9e-7).

We tested the impact of varying the intrinsic CN parameters for all SWs by plotting them on the same MDS plot. The effect of the seed weight on the end weight landscape was larger than those of the intrinsic CN parameters (Figure 12B), but it is clear that both categories of seed factors had a major impact on learning outcome. But irrespective of seed weight or intrinsic seed factors, the learning process always resulted in a selection of correlated sensors to become the end weight winner synapses (Figure 13). The fact that the learning worked in a similar fashion across a range of settings of intrinsic parameters for defining the intrinsic dynamics of the CN shows that the model architecture has some degree of robustness, i.e., it is independent of having precise constraints on neuron intrinsic behavior and will work similarly across a population of neurons with different intrinsic dynamics. Such differences within a CN population may be important to identify different subsets of correlated sensors in the learning process, and hence creating a diversification in the tuning of across the population of cuneate neurons, similar to the one observed *in vivo* (Jörntell et al., 2014).

## Discussion

Using an integrated model of cuneate nucleus neurons, based on the present *in vivo* whole cell recording data, and a bionic tactile sensor system, this study investigated what form of representations the brain could automatically learn from tactile interactions with the world. At the single neuron level, the CN learning resulted in the identification of sets of functionally correlated sensors (Figures 5, 13). By reducing or eliminating the synaptic weights of other sensors, this process essentially equals a dimensionality reduction of the sensory space at the single neuron level. At the population level, each CN, as defined by its unique seed factors, gradually identified different sets of functionally correlated sensors (Figures 7–9, 13). As a consequence, the learning process induced decorrelations in the synaptic responses of the different CNs to the same stimulus as well as for the individual CN to different stimuli (Figures 7, 8).

Such decorrelated synaptic responses is a key requirement for a population of cuneate neurons to be able to segregate the high-dimensional tactile sensory space, where each sensor potentially represents a unique dimension (Spanne and Jörntell, 2013), into specific projections (Jörntell et al., 2014). These projections are defined by the types of sensory experiences made (Figure 11) and the physical-mechanical effects involved (see Introduction). Hence, the representation of the physical world that naturally unfolded given the neurophysiological constraints of the cuneate neurons *in vivo* (Figure 1) was a utility-based decomposition of the sensory space. Thereby, the architecture of the cuneate nucleus network (Figure 3) would help the brain focusing to focus on the statistically recurring elements of experienced tactile sensory activation patterns. It follows that an early rich variation of tactile sensory experiences (Pape et al., 2012; Loeb and Fishel, 2014), such as achieved by motor babbling in the infant (Forssberg et al., 1995; Blumberg et al., 2013), is important for the brain because it would be needed to form representations with a sufficiently high dimensionality.

### Robustness of the learning process suggests little dependence on assumptions

In order to address a breadth of issues that related to the representation of haptic input features in the cuneate nucleus neurons (Jörntell et al., 2014) and their intrinsic properties, but which are too complex to be resolved by experimental recordings, we needed to model the cuneate neurons. Whereas, the model system naturally could have featured a higher number of sensors or a larger number of contexts across which the generalization capability was tested, we find no reason to believe that would have altered the principles reported here. An important component of the model was the learning process that we envisage to be one of the earliest developmental steps toward a functional tactile neural system. Although there are several indications that the PA synapses on the cuneate neurons are extensively plastic at least during some phase of development (Bengtsson et al., 2013) and following removal of PA inputs (Kambi et al., 2014; Mowery et al., 2014), the direct synaptic mechanisms underlying the plasticity have not been explored. Therefore, in order to model this learning process, we used an already validated idea of a calcium activity-dependent mechanism (Helias et al., 2008), which to some extent can be regarded a general synaptic plasticity mechanisms of neurons. It is in principle a variant of the BCM learning rule (Bienenstock et al., 1982) for which we obtained specific data from the biological cuneate projection neurons to approximate how their internal calcium signal can be expected to vary over time and with the input. In comparison with a previous study of how calcium-dependent STDP varies across a range of parameter settings (Graupner and Brunel, 2012), our approach had a less steep rising phase in the postsynaptic calcium response, which can be expected to dampen the dependence of our model on exact spike timings and instead make it rely to a larger extent on the temporal variation in the intensity of the synaptic activation (Graupner et al., 2016) and how it correlated with the overall activity of the postsynaptic neuron. Naturally, the actual cuneate learning process is likely to differ from ours in many details but the fundamental properties of our model, i.e., an extreme dimensionality reduction in each neuron and the identification of different correlated sets of sensors by the population of CNs (Figure 7), were remarkably robust to manipulation of a wide variety of neuronal parameters that influenced the learning process (Figures 12, 13). It can also be noted that after learning, our CNs had synaptic weight distributions that were similar to adult cuneate neurons (Figure 5C and Figure S3) (Bengtsson et al., 2013) as well as response dynamics that qualitatively captured the dynamics recorded *in vivo* (Figure 6D, Figure S1).

Plasticity in inhibitory synaptic inputs could in principle help improving segregation of inputs (Garrido et al., 2016). Our analysis of the biological inhibitory synaptic inputs in the cuneate nucleus did not overtly support a specific role in such function, though. The recorded inhibitory interneurons in the cuneate nucleus were activated from wide receptive fields and in the projection neurons inhibition was found to originate from similar wide receptive fields, with relatively uniform inhibitory synaptic weights (Figures 1C,D). This is in contrast to the excitatory synaptic inputs from PAs, which in cuneate projection neurons have been found to have strongly differentiated weights and hence suggestive of an extensive learning history in these synapses (Bengtsson et al., 2013). In our CN model, the plasticity in the inhibitory synapses instead assumed the role of activity balancing, i.e., ensuring that the excitable calcium responses did not go out of bounds (neither upwards, nor downwards), which was an important function to ensure stable, gradual learning (cf. Figure 10).

### Advantages of the representational format and of the architecture

The utility-based decomposition of the tactile sensory space learned with our CN architecture is likely to correspond to an early level acquisition of a representation of the experienced, and therefore useful, haptic input features, which were previously found to be represented in cuneate neurons *in vivo* in adult animals (Jörntell et al., 2014). Being based on physical-mechanical effects (Hayward, 2011; Hayward et al., 2014), correspondences between the haptic input features and sensory information from other modalities such as vision and hearing can be expected to be abundant. Hence, this type of tactile representation would provide a basis for rich cross-modality representations of rules of object interactions that can be learnt by the higher level centers of the brain to conceptualize the external world (O'Regan and Noe, 2001). This model does not contradict the possibility that the activity of individual cuneate neurons or neurons of the somatosensory cortex could correlate with physical parameters such as edge orientation or texture or even adaptation rate, but indicates that the underlying organizational and coding principle is likely to be more intricate than that.

These principles are potentially portable to AI and robotic systems, which faces challenges of how to design sensor systems to support more general functionality in the interactions with the external world (Brooks, 1991; Davis et al., 1993; Service, 2014). AI systems are generally designed to deliver performance on specific tasks, such as the game of Go (Gibney, 2016), where they deliver impressive performance if given very large amounts of training data (Lecun et al., 2015). However, they seem to be lacking the wide versatility or generalization capability of biological systems (Nguyen et al., 2015; Athalye and Sutskever, 2017), which could be related to the known problems of classical pattern recognition systems (Spanne and Jorntell, 2015) and which the current alternative mode of representation of sensory input data may help resolving. These technical systems also in general suffer from a strong dependence on the initial weight configuration (Glorot and Bengio, 2010; He et al., 2015). This dependence can be eliminated by specifically dedicated computational mechanisms calculating appropriate initial weights or by other computationally intensive methods (Williams and Hinton, 1986; Földiak, 1990; Glorot and Bengio, 2010), which, however, may not be available to the brain. Our architecture, featuring a set of neuronal auto-regulatory mechanisms and self-stabilizing learning, provide potential solutions also for these challenges within DNN and AI systems.

## Concluding remarks

A main advantage of representing tactile experiences by decomposing them into vector components of their constituent physical effects, rather than by pattern recognition applied to pixelated sensory data directly, is the powerful generalization possibilities (Spanne and Jorntell, 2015). With this mode of representation, the brain could in principle learn to identify the extent of the space of the theoretically possible physical effects within a short time using just a few types of disparate skin-object interactions and later subsequently learn to interpolate new points within this space at a gradually higher resolution. A major issue for future studies is to explore if brain processing operates in terms of such vector components also globally in the neocortex.

## Author contributions

UR, AS, and HJ designed the model. FB and HJ performed the patch clamp recording experiments and analyzed the biological data. UR, AM, and CO developed and experimented the bionic finger for electrophysiological and translational bioengineering studies. UR, AS, AM, CO, and HJ designed the analysis of the model data. UR, CO, AM, and HJ wrote the paper.

### Conflict of interest statement

The authors declare that the research was conducted in the absence of any commercial or financial relationships that could be construed as a potential conflict of interest.
