# Supplementary material for: Intracellular Dynamics in Cuneate Nucleus Neurons Support Self-Stabilizing Learning of Generalizable Tactile Representations
Source: Front Cell Neurosci. 2018 Jul 31;12:210. doi: 10.3389/fncel.2018.00210 (PMC6079306; doi:10.3389/fncel.2018.00210)
Supplement: Supplementary file 1 [file Data_Sheet_1.pdf]

# Intracellular dynamics in cuneate nucleus neurons support self-stabilizing learning of generalizable tactile representations

## Supplementary Data

U. B. Rongala, A. Spanne, A. Mazzoni, F. Bengtsson, C. M. Oddo, H. Jörntell.

**Supplementary Tables 1 to 2**

**Supplementary Figures 1 to 3**

| Stimuli | | Experiment Protocol |
|---|---|---|
| Naturalistic Texture | Latex | Passive touch protocol: The sensorized fingertip is fixed and the texture slides along the surface of the finger. The sequence of stimuli movements involved are *contact on, followed by immediate onset sliding and contact off*. |
| | Glass | |
| | Velvet | |
| Shapes | Shaft with 5 mm radius | Active touch protocol: The fingertip is mechanically indented against probes with two different shapes. The sequence of fingertip movements including *indentation and retraction*. |
| | Shaft with 20 mm radius | |

**Table S1. Training stimuli for the artificial fingertip.**

| A | B | C | $C_m$ | a | b | c | d |
|---|---|---|---|---|---|---|---|
| 0.04 $s^{-1}V^{-1}$ | 5 $s^{-1}$ | 140 $Vs^{-1}$ | 1 F | 0.02 $s^{-1}$ | 0.2 | -65 mV | 8mV |

**Table S2. Izhikevich model parameters used in the neuromorphic sensors.**

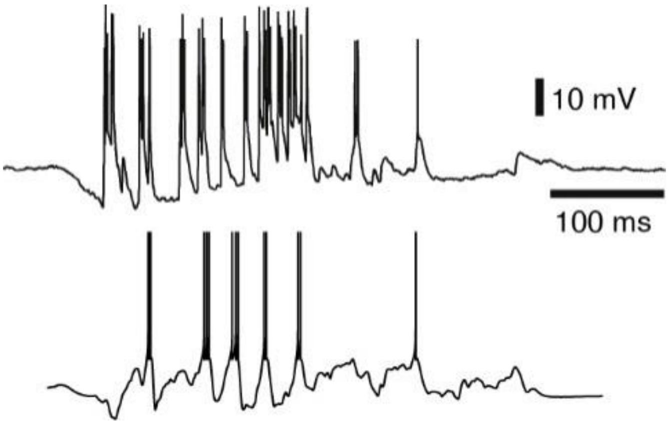


**Figure S1.**

**Response dynamics in recorded and modelled CNs.**

Comparison of responses evoked by dynamic, light indentations (‘touch’) in an *in vivo* cuneate projection neuron and in a modelled CN. Notably, even though the approximate stimulus durations were the same, the exact patterns of PA afferent synaptic inputs were not. Still, the responses share many overall dynamic features such as an early inhibition, spike bursts and very rapid transitions between spike AHPs and subsequent bursts.

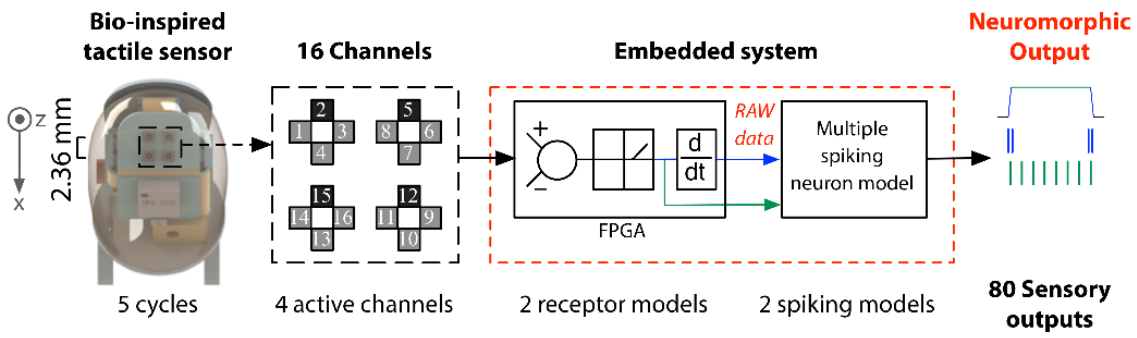


**Figure S2.**

**Spike generation from in the sensors of the bionic fingertip.**
Sensors of the bionic fingertip and their alternative modes of generating spike outputs. Further description of its transduction process is in the Methods and in [2].

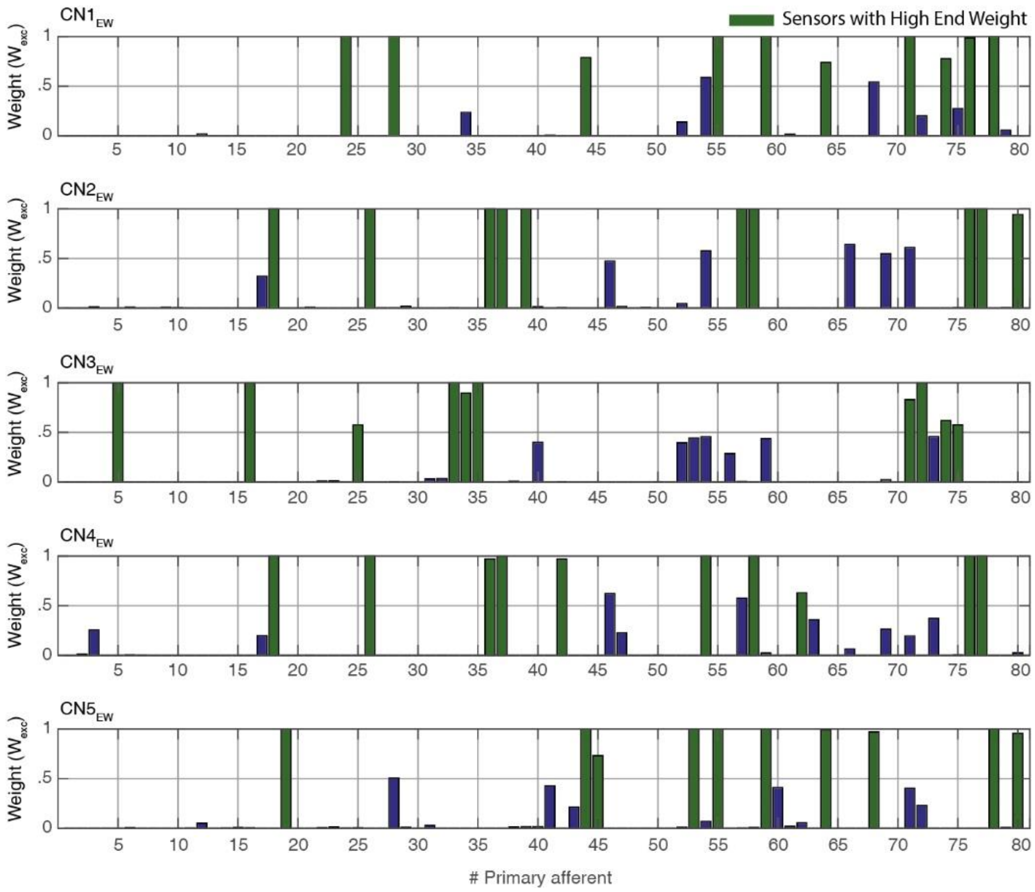


**Figure S3. End weight landscapes for the five different seed weights.**

End weight landscapes for the five different seed weights. Green bars in each panel indicate the end weight winners used for calculating correlation index differences in Figure 5D.

## References


1. Bengtsson F, Brasselet R, Johansson RS, Arleo A, Jorntell H (2013) Integration of sensory quanta in cuneate nucleus neurons in vivo. PLoS One 8: e56630.
2. Oddo CM, Mazzoni A, Spanne A, Enander JM, Mogensen H, et al. (2017) Artificial spatiotemporal touch inputs reveal complementary decoding in neocortical neurons. Scientific Reports 8: 45898.
